# Genetic and neuro-epigenetic effects of divergent artificial selection for feather pecking behaviour in chickens

**DOI:** 10.1186/s12864-024-11137-w

**Published:** 2024-12-19

**Authors:** Elske N. de Haas, Fábio Pértille, Joergen B. Kjaer, Per Jensen, Carlos Guerrero-Bosagna

**Affiliations:** 1 Department of Veterinary Science, Animals in Science and Society, Utrecht University, Utrecht, The Netherlands; 2 Behavioural Ecology Group and Adaptation Physiology Group, Department of Animal Sciences, Wageningen University, Wageningen, The Netherlands; 3 Escola Superior de Agricultura “Luiz de Queiroz”, São Paulo, Brazil; 4 IFM Biology, Avian Behaviour Physiology and Genomics Group, Linköping University, Linköping, Sweden; 5 Federal Research Institute for Animal Health, Celle, Germany; 6 Department of Veterinary and Animal Sciences, University of Copenhagen, Copenhagen, Denmark; 7 Physiology and Environmental Toxicology Program, Department of Organismal Biology, Uppsala University, Uppsala, Sweden

**Keywords:** Feather pecking, Chickens, Genomics, DNA methylation, Single nucleotide polymorphisms, Copy number variations, Quantitative Traits Loci, Artificial selection

## Abstract

Feather pecking (FP) is a repetitive behaviour in chickens, influenced by genetic, epigenetic, and environmental factors, similar to behaviours seen in human developmental disorders (e.g., hyperactivity, autism). This study examines genetic and neuro-epigenetic factors in the thalamus of chickens from lines selected for seven generations for high or low FP behaviour (HFP or LFP). We integrate data on Differentially Methylated Regions (DMRs), Single Nucleotide Polymorphisms (SNPs), and Copy Number Variations (CNVs) in this controlled artificial selection process. Significant differences in behaviour, immunology, and neurology have been reported in these lines. We identified 710 SNPs in these lines that indicate new potentially important genes for FP such as *TMPRSS6* (implicated in autism), and SST and *ARNT2* (somatostatin function). CNV were the omic level most affected during selection. The largest CNVs found were in *RIC3* (gain in HFP) and *SH3RF2* (gain in LFP) genes, linked to nicotinic acetylcholine receptor regulation and human oncogenesis, respectively. Our study also suggests that promoters and introns are hotspots for CpG depletion. The overlapping of the omic levels investigated here with data from a public FP Quantitative Trait Loci (QTL) database revealed novel candidate genes for understanding repetitive behaviours, such as *RTKN2*, associated with Alzheimer’s disease in humans. This study suggests CNVs as a crucial initial step for genomic diversification, potentially more impactful than SNPs.

**Supplementary Information:**

The online version contains supplementary material available at 10.1186/s12864-024-11137-w.

## Background

Behaviour can be seen as the output of the brain’s orchestra, influenced by its chemistry and functionality. Factors that change gene expression in specific brain regions can make individuals predisposed to develop certain behaviours [1]. In humans, neurodevelopmental disorders are generally diagnosed and categorized based on the occurrence of specific behavioural patterns combined with genetic predisposition [2]. In farm animals, artificial selection on behaviour traits is well known to effectively produce divergent phenotypic patterns in only a few generations [3–6].

Some behavioural problems in animals appear to be similar to those observed in humans in what concerns their aetiology, phenomenology and underlying neurobiology. One example is FP in chickens, which displays similarity with body-focused repetitive behaviour (BFRBs) in humans with high somatic activity [7, 8]. While chickens performing FP peck the feathers of other chickens, humans performing BFRBs are generally targeting their own body. Examples of compulsive grooming habits in humans that fall under the BFRBs criteria are hair pulling (trichotillomania), skin picking and nail biting [9]. Although some BFRBs can be reduced with selective serotonin reuptake inhibitors (SSRI) [10], more severe and damaging manifestations of these BFRBs seem to be difficult to inhibit or stop, as they appear compulsive and perseverating [11]. Similarly, in chickens FP behaviour can also be reduced with drugs that interfere with the process of serotonin reuptake [12, 13].

Genetic predisposition together with stressful conditions, particularly during early life, can result in the development of BFRBs [14]. In chickens, early life deprivation of environmental resources can result in the emergence of FP in young [15, 16] and adult individuals [17–19]. FP is generally seen as a repetitive behaviour, perhaps redirected from foraging and exploration and typically triggered under stressful conditions [20], such as crowded and stimulus poor environments. FP occurs particularly in genetically predisposed anxious individuals [21, 22], even if a population is highly homogenous in terms of genetic background [23–25]. Previous research in a line selected for FP behavior has shown informative features about the genetic basis of this behavior, such as the existence of a group of animals exhibiting severe (hyperactive) FP, a suggested allele associated with this severe FP which can be eliminated in two rounds of selection, and the overall polygenic nature (of highly interrelated genes) of FP behavior [26]. Additionally, factors other than genetic background can influence the predisposition to develop FP. Among these non-genetic factors, epigenetic modifications in brain regions appear to be of relevance to understand the aetiology of behavioural disorders [27]. In the present paper, we explore in chickens the genomic and epigenomic divergence occurring in experimental lineages subjected to controlled selection for high or low FP behaviour.

Epigenetic mechanisms involve chemical modifications to the DNA itself or to protein complexes that pack the DNA (histones). These chemical modifications can survive cell divisions and are involved in the long-term, tissue-specific ability to regulate gene expression [28]. One of these epigenetic modifications is DNA methylation, which corresponds (mainly) to the enzymatic addition of methyl groups to cytosine neighbouring guanines (5’ to 3’), dinucleotides known as CpG sites [29]. DNA methylation is a key regulator of gene expression in the brain and related to neurological disorders such as schizophrenia, depression, addiction [30], autism [31], anxiety [32], and hyperactivity disorder [33]. Changes in whole-brain gene expression patterns have also been observed in chickens performing FP compared to control or neutral chickens [34–36]. These differentially expressed genes are involved in neurotransmission and immunology [36]. Similarly, a more recent study finds brain gene expression changes related to cholinergic signalling, channel activity, synaptic transmission, and immune response [37]. A follow up gene-gene interaction network analysis found enrichment of *KLF14* binding sites in FP differentially expressed genes in the brain, and that a genetic variant in the proximity of *KLF14* binding sites associate with this differential expression, with suggested consequences for T-cells brain levels [38]. Immunological alterations in relation to FP are not restricted to the brain, as they have also been reported at the organismal level [39]. Interestingly, immunological alterations have also been linked to OCDs in humans [40].

Both genetic and neuro-epigenetic factors were investigated here in the thalamus of chickens from the 18th generation of lines initially selected for 7 generations for either high or low FP behaviour and later maintained. Previously, it was observed that both gene expression and methylomic changes emerge in the hypothalamus of chickens after five generations of divergent selection for high or low fear of humans [41]. The thalamus is a brain region involved in the regulation of stress response in chicken [42]. Our FP selection model has generated chickens with marked differences in behaviour, immunology, and neurology [13, 39]. In the present analyses, we employed a newly developed method in which methylated immunoprecipitation (MeDIP) [43] is coupled to genotyping-by-sequencing (GBS) [44] to assess both genomic differences (SNPs and CNVs) and methylomic variation in the same genomic fraction of the individuals investigated. Omic differences in DMRs, SNPs and CNVs were investigated between chickens from these selection lines. Additionally, we combined these data with a public FP QTL database (QTLdb) to perform comparative genomic analyses. This is the first study that integrates data on DMRs, SNPs and CNVs during a controlled process of artificial selection to understand the underlying genomic and epigenomic patterns of divergence.

## Methods

### Animals and housing

Ethical approval as given by the Central Authority for Scientific Procedures on Animals according to Dutch Law (no: AVD104002015150), as part of another study [45]. In this study we compared chickens from lines divergently selected on FP behaviour. The founder line was a synthetic random-bred White Leghorn population initially kept at the Danish Institute of Agricultural Sciences, today part of Aarhus University. From this founder line, two separate lines were established and divergently selected for 7 generations for high (HFP line) or low (LFP line) levels of FP behaviour. Phenotyping for the selection was based on direct behavioural observation of the chickens kept in floor housing in small groups, within an observational range of 180 min at 68 weeks of age [46]. The selection trait was the frequency of FP, expressed as the number of bouts per hour. This trait included gentle FP (soft nibbling and pecking, without causing damage to the recipient) and severe FP (forceful pulls and pecks, leading to pulled out feathers and plumage damage to the recipient). Pecks directed to the same chicken to the same body part within 5–10 s were combined to one bout, thus lending relatively more selection pressure to severe FP, which often occurs in short series, rather than to gentle FP, which often occurs in long series [47]. After the 7th generation, the lines were still kept separate and reproduced each year by random mating. The selection resulted in a consistently higher FP rate, with higher number of bouts and pecks, and a higher frequency of birds performing FP, in the high HFP line compared to the low LFP line, generation after generation [46]. Then, these lines were maintained separately until animals from the 18th generation were employed in the present experiment. These were hatched and housed at the experimental research facility of Wageningen University and Research, The Netherlands. Incubation, housing and management details were described elsewhere [45]. Dams were housed at the age of 50 to 54 weeks with roosters from the same line. In both lines, 40 dams and 5 roosters were used. Ten pens were used, where each pen housed one rooster with 8 dams per line, i.e. either HFP or LFP chickens in one pen. Collected eggs per pen could be backtracked to the sire, but not the specific dams within the same pen.

### Sample collection

At 8 weeks of age, two female pullets per sire were randomly taken for DNA-analysis, in total 20 pullets, 10 per line. The pullets were taken individually from their home pen and killed by decapitation. Brains were dissected, frozen in liquid nitrogen, labelled numerically without indication of the lineages, stored, and shipped to Linköping University for further analysis. The code for the samples was only revealed after bioinformatic pre-processing, at the moment of performing downstream statistical genomic comparisons.

### DNA extraction and preparation of sequencing libraries

Brains were thawed immediately before DNA extraction, which was performed on 30–40 mg of the homogenized thalamus with the D-Neasy Blood & Tissue Kit (Cat. No. 69504) as per the manufacturer’s instructions. After extraction, the quality of the DNA was evaluated by electrophoresis using 1.5% of agarose gel and Nanodrop (ND-1000 spectrophotometer- (Saveen Werner). Then individual DNA samples were used for sequencing library preparation, performed using a combination of the GBS [44] and MeDIP [43] methods. This combination of methods has been previously applied in chickens [43, 44, 48, 49].

Briefly, the genomic DNA was digested with *PstI* Restriction Enzyme (NEB, Ipswish, USA), leading to a reduced genome of approximately 2% of the complete genome and enriched short reads for Illumina sequencing (200–500 bp). A barcode adapter (for interindividual identification) and a common adapter for Illumina sequencing were ligated at both ends of the digested DNA fragments [50]. With the barcodes, GBS enables creation of a sequencing library with pooled DNA of different individuals [51, 52]. After ligation, samples are pooled and cleaned up to eliminate primer dimers and unbound adapters [51, 52]. A 100 ng portion of this pooled DNA is then amplified by PCR, corresponding to the genetic fraction of the genome of the individuals (named Input library). Another 5 µg portion of the pool was used for an anti-methyl-cytosine antibody (2 µg µl^− 1^; catalogue number C15200006, Diagenode, Denville, NJ, USA) immunoprecipitation. This antibody preferentially captures the methylated fraction of the pooled DNA. This methylated fraction is then amplified by PCR and finally cleaned up same as the input. Both cleaned libraries (input and methylated fraction) are then paired-end sequenced on the IlluminaHiSeq2500 platform using 125 bp length reads. For a complete protocol describing the technique refer to Rezaei et al. [53]. Sequencing was performed at the facilities of the SciLifeLab (SNP&SEQ, Solna, Sweden).

### Bioinformatic analyses

Data from the sequenced libraries were processed by CASAVA (Illumina) by converting “.bcl” (base calls) to “.fastq” format, as compatible to other programs for reads alignment. Quality of short reads were checked with FastQC v.0.11.33. Reads for SNPs, CNVs and DMRs were aligned to the chicken reference genome (Gallus_gallus-5.0/galGal5, RefSeq: GCF_000002315.4, NCBI) available at the time using default parameters for Bowtie2 tool v.2-2.3.4.2 [54]. The coverage depth of each sequenced file was determined using Samtools version 1.19 with the “depth” option.

From the sequences generated by input sequencing, SNP calling was executed by Tassel v.3.0 [51], using default TASSEL-GBS Discovery Pipeline. Criteria for inclusion were at least 2% for minimum minor allele frequency (mnMAF), 20% of minimum taxon/ sample coverage (mnTCov) and 70% for minimum site coverage (mnScov). SNPs that passed the filtering criteria were selected for an allele-based association test using Plink software v2 (2009 Shaun Purcell, GNU General Public License), in which HFP was compared to LFP (set as control). For this, we used the --assoc command, calculating chi-squared statistics to evaluate associations between genotypes and divergent selection lineages. Additionally, we conducted permutation testing (--perm) to obtain empirical p-values. We then applied multiple-test correction using Benjamini-Hochberg false discovery rate (FDR) with an adjusted P-value ≤ 0.05 to select significant SNPs. A visual representation of the distribution of the individuals based on the SNPs observed among the selection lines was performed by PCA cluster analysis. For this, the s.class function from the adegenet R package was used to scatter plot a factorial map of the two first PCs of the individuals clustered in two groups (HFP and LFP). The ellipses display the distribution of the individuals from each experimental group Based on the PC values. An Archaeopteryx tree [55] was then plotted using a cladogram generated by Neighbor Joining (NJ) distance matrix generated by the Tassel v.3.0 software.

For the Fst and Tajima’s D analysis, whole-genome sequences of the sampled population were aligned to the chicken reference genome, and the genome was indexed using samtools. Per-site theta values were estimated using ANGSD tools in bash environment, followed by the computation of window-based statistics. Specifically, Tajima’s D values were extracted for further analysis. Next, the VCF files containing SNP data were processed using R. We used the vcfR, hierfstat, and pegas packages to convert VCF data into genind format and further into a hierfstat format suitable for computing population genetics statistics. The global Fst was computed for all SNPs, providing a measure of the genetic differentiation between the two predefined groups. For individual SNP analysis, we derived the allele frequencies and calculated Fst values for each SNP. We then matched these Fst values with corresponding Tajima’s D windows and aggregated the values to produce average statistics for each SNP. Subsequently, SNPs were classified based on their Fst and Tajima’s D values using the following criteria: Fixed Allele: Fst = 1; Positive Selection: Fst > 0.25 & Tajima’s D < 0; Balancing Selection: Fst > 0.25 & Tajima’s D > 0; High FST - Neutral Tajima: Fst > 0.25 & Tajima’s D = 0; Negative Selection: Fst < 0.05 & Tajima’s D < 0; Possible Population Structure/Expansion: Fst < 0.05 & Tajima’s D > 0; Neutral: 0.05 ≤ Fst ≤ 0.25; Non-Selected: Fst < 0.05 with no corresponding Tajima’s D data; High FST - No Tajima’s Data: Fst ≥ 0.05 with no corresponding Tajima’s D data; Shared Neutrality: All remaining SNPs. A scatter plot was constructed using ggplot2, plotting the Fst values against the Tajima’s D values.

To investigate allele differences between the lines, we first divided the SNP table into two, HFP and LFP, and we set the allele frequency equal to 0 or 1 to filter only homozygous genotypes. Subsequently, we merged the tables in order to assess which allele differences (only homozygous genotypes) were found between the lines. We compared T↔ C transitions between the lines as these allele changes primarily occur in CpG sites, which are depleted in vertebrate genomes due to their hypermutability compared to other dinucleotides [56]. Then, we identified the neighbouring bases of these T/Cs to discern whether these nucleotides belonged to a CpG dinucleotide in the reference genome. We accessed sequence information of specific genomic regions of the chicken genome using BSgenome::getSeq in R.

QTL data was obtained from the chicken QTLdb release54 (https://www.animalgenome.org/cgi-bin/QTLdb/index; chickenGG5.gff.gz*)* and the SNP overlapping test with QTLs for FP was performed using the GenomicRanges package. These analyses were performed within R environment and the packages were downloaded from the Bioconductor repository.

For the CNV and DMR calling, reads from the input and the methylated sequenced libraries were respectively used. First, we used Stacks v.1.39 for data de-multiplexing [57] and for maintaining quality trimmed reads for the sequenced libraries. For CNV calling, the aligned sequence files (.bam) of each individual (from each treatment) were merged into unique files. The “view” option from Samtools v.1.3.14 [58] was used to generate a “hit” file from each unique file containing the coverage information for each base pair sequenced from each treatment. This “hit” file was then used for CNV calling by the CNV-Seq tool [59] across the chicken genome using default parameters. While for DMR call, following read alignment, all analyses were performed using bioinformatics packages from the ‘R’ Bioconductor repository. The BSgenome.Ggallus.UCSC.galGal5 package was uploaded as the reference genome. The MEDIPs R-package was used for basic data processing, quality controls, normalization, and identification of differential coverage. In order to avoid possible artefacts caused by PCR amplification, MEDIPs allows a maximum number of stacked reads per genomic position. This is done by using a Poisson distribution of stacked reads genome-wide. The default parameter of *P* < 0.001 was used as the threshold for the detection of stacked reads. The reads that passed this quality control were then standardized to 100 bp by extending smaller reads to this length (100 bp is the paired-end read size generated by the Illumina HiSeq platform). The genome was divided into adjacent windows of 300 bp length, which was the expected average length of contigs generated by our GBS approach, as well as the program default. MeDIP-seq data were transformed into genome-wide relative methylation scores by a CpG-dependent normalization method [60]. This normalization is based on the dependency between short-read coverage and CpG density at genome-wide windows [61] and can be visualized as a calibration plot. A calibration plot was generated using one of the 10 individuals that passed the cut-off index to generate a coupling set (object that groups information about CpG density genome-wide). Based on this, a threshold for a minimum sum of counts across all samples per window was defined (minRowSum = 10). Sequencing data for each individual were then assigned to one of the experimental groups (HFP and LFP) and differential coverage (i.e. differential methylation) was calculated between the two lines. Adjacent windows showing significant change were then merged to generate the DMR obtained. For this, the default value of 1 was used within the function MEDIPS.mergeFrames, allowing the neighbouring significant windows to be merged with a 1 bp gap between them.

The genomic coordinates of the different assays (SNPs, DMRs, CNVs, and QTLs) were annotated against the chicken reference genome (BSgenome.Ggallus.UCSC.galGal5) using the annotatePeak function from the ChIPseeker package [62] in R. In this function, we used the gg_txdb (as the transcript metadata) from the GenomicFeatures package and org.Gg.eg.db package as the annotation database for the chicken genome. We used g: Profiler as the web server for functional enrichment analysis [63]. Specifically for significant SNPs located in exonic regions, the coordinates were converted from galGal5 to galGal6 using the LiftOver tool from GenomeBrowser (https://genome.ucsc.edu/cgi-bin/hgLiftOver) and annotated with the Variant Effect Predictor (VEP) tool [64], which included calculation of SIFT scores to predict the potential functional impact of amino acid substitutions for these SNPs. SIFT scores range from 0 to 1, where values closer to 0 indicate a higher likelihood of a deleterious effect on protein function, while scores closer to 1 suggest the substitution is likely to be tolerated. Description of the SNP-related genes were further explored by Uniprot and QuickGO online genomic information databases.

Finally, we performed overlap tests between the genomic range coordinates of the significant SNPs, CNVs, and DMRs found in our study, and publicly available QTLs for FP. This was done using the ChIPpeakAnno package from R, which employs a hypergeometric test (hyperG) as the default parameter. We then plotted a Venn Diagram with the “makeVennDiagram” function of the package. The idea was to identify if the genetic and epigenetic variants identified here located within genomic regions previously reported to influence FP.

Finally, the repeat mask annotation data for the chicken genome galGal6 (rmsk.galGal6.Nov2018.rds) was retrieved using the AnnotationHub R package. To standardize the repeat mask annotation, we converted the galGal6 repeat mask coordinates to galGal5 with the LiftOver function from rtracklayer R package, using the galGal6ToGalGal5.over.chain.gz index from UCSS as input. After that, we used the converted RepMask intervals to detect its overlaps against the coordinates of our assays (SNPs, CNVs, and DMRs), using functions from the GenomicRanges and dplyr R packages. The frequency of each feature was calculated and then plotted using ggplot2 in R.

## Results

### Sequencing alignment and SNP analyses

**Fig. 1:**
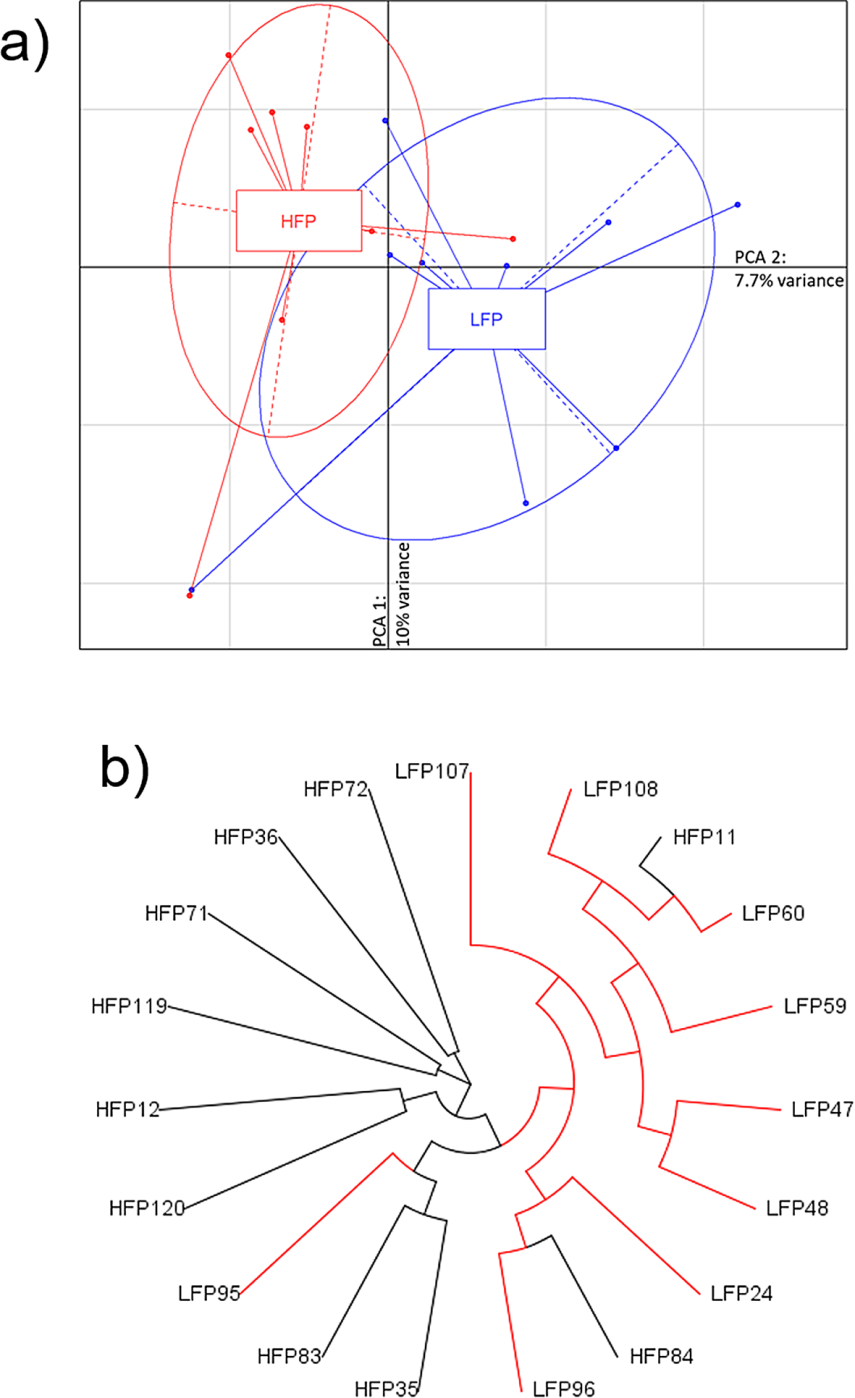
Genetic separation of individuals in each lineage (9 HFP and 7 LFP), based on 76,414 SNPs remaining after filtering (sample call rate ≥ 20%; loci call rate ≥ 70%), employing **a**) PCA or **b**) Neighbour Joining distance

The average sequencing coverage was 16.2 ± 11.2 X for the GBS. A total of 100,523 SNPs was identified among all 19 sequenced individuals using Tassel (default parameters). After a sample call rate ≥ 20% and loci call rate ≥ 70%, 16 individuals remained (9 HFP and 7 LFP) and 76,414 SNPs were kept for further analysis. To confirm the representativity of our SNP panel, we calculated the recombination rates (r^2^) across SNP pairs. We found a low average r^2^ (0.038) and median r^2^ (0.011), with 75% of r^2^ values falling between 0.0038 and 0.0437 (Additional file 1 Fig. S1). This indicates low LD, supporting the independence of our SNPs. Conversely, we found only a few SNP pairs with high LD; these instances are limited and likely represent specific, tightly linked regions. The 76,414 SNPs were then used to perform a PCA which resulted in two eigenvalue factors: PC1 explaining 10% and PC2 explaining 7.7% of the variance (Fig. 1a). Additionally, Neighbour Joining (NJ) distance analysis based on SNP similarity generated two genetically different clusters of branches: one for HFP and one for LFP chickens (Fig. 1b). Figure 1a and b both show the separation of the individuals between the selection lines, in which some individuals did not cluster in accordance to their predefined groups: these were LFP95, HFP84 and HFP11.

The SNPs obtained here are not based on phenotypic (FP) differences, but rather on lineage differences (HFP and LFP). However, in order to obtain hints of SNPs that could be related to the phenotype, we performed an Fst analysis to identify SNPs with strong signals of genetic differentiation between the lineages. We computed the global Fst and Tajima’s D for all SNPs to estimate the level of genetic differentiation of the population, which contains the two predefined groups HFP and LFP. We found values of Fst = 0.045 and Dst = 0.017, which indicates genetic differentiation and population structuring/expansion, confirming a genetic effect of the selection for high/low FP in our population. Additionally, we derived the allele frequencies and calculated Fst values for each SNP. These Fst values were matched with corresponding Tajima’s D windows and aggregated the values to produce average statistics for each SNP. The classification of SNPs in relation to their Fst and Tajima’s D values are shown in Additional file 1 Fig. S2 and Additional file 2, Table 1. The Fst analysis identified 22 SNPs showing strong signals of genetic differentiation between HFP and LFP lines, located across various genomic regions, including promoters, introns, exons, and distal intergenic regions. Many of these variants are in or near genes associated with neurodevelopment, stress response, and cellular signalling. Notably, several SNPs were located in promoter regions near genes such as *TMPRSS6* (transmembrane serine protease 6) and *PPP2R5C* (protein phosphatase 2 regulatory subunit B’gamma). Other SNPs were located in genes with neurological function, such as *SST* (somatostatin) and *ARNT2* (aryl hydrocarbon receptor nuclear translocator 2).

**Fig. 2:**
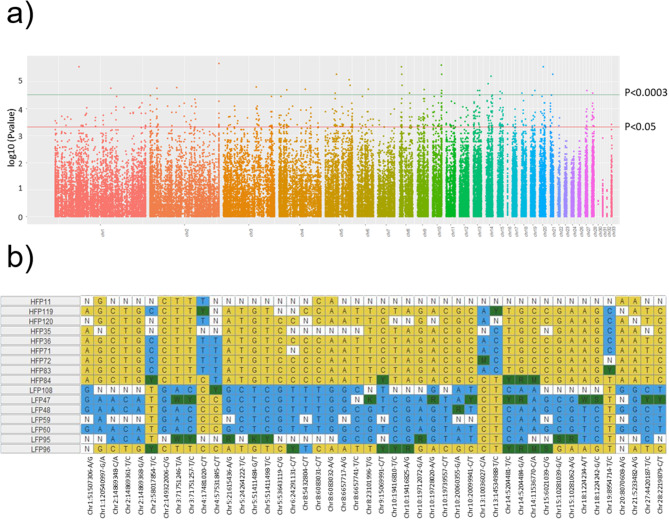
**a**) Manhattan plot depicting the SNPs that crossed the adjusted P-values of *p* ≤ 0.05 (711 SNPs; red line) and *p* ≤ 0.0003 (46 SNPs; green line) after filtering by Benjamini-Hochberg false discovery rate (FDR); **b**) Representation of the allelic differences across the individuals on the HFP and LFP lineages obtained from 46 SNPs with *p* ≤ 0.0003

**Fig. 3:**
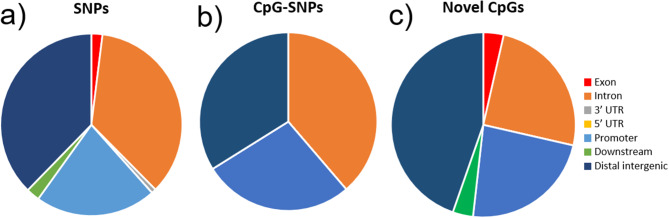
Functional annotation of **a**) SNPs, **b**) CpG SNPs, and **c**) Novel CpGs found between the animals in the LFP and HFP lineages

Next, we performed an allele-based association test followed by permutation testing comparing the HFP and LFP based on the 74,759 SNPs that remained after quality control adjustments, including setting 20,622 heterozygous haploid genotypes to missing and excluding 1,655 SNPs with insufficient genotyping data, resulting in a final genotyping rate of 0.82. These SNPs were further filtered based on the Benjamini-Hochberg false discovery rate (FDR), using an adjusted P-value ≤ 0.05. This resulted in 711 significant SNPs between the HFP and LFP individuals (Fig. 2a, see Additional file 2 Table S1), which were mainly located at distal intergenic regions (37.7%) followed by intronic regions (35.6), in promoters (21.5%), downstream of genes (2.4%) and exonic regions (2.0%) (Fig. 3a). It is worth highlighting that all the 22 SNPs found with strong signals of genetic differentiation among the lineages in the Fst analysis have also passed the GWAS significance threshold. For visualization purposes, in Fig. 2b we show the allelic differences of 46 SNPs with *p* ≤ 0.0003.

**Fig. 4:**
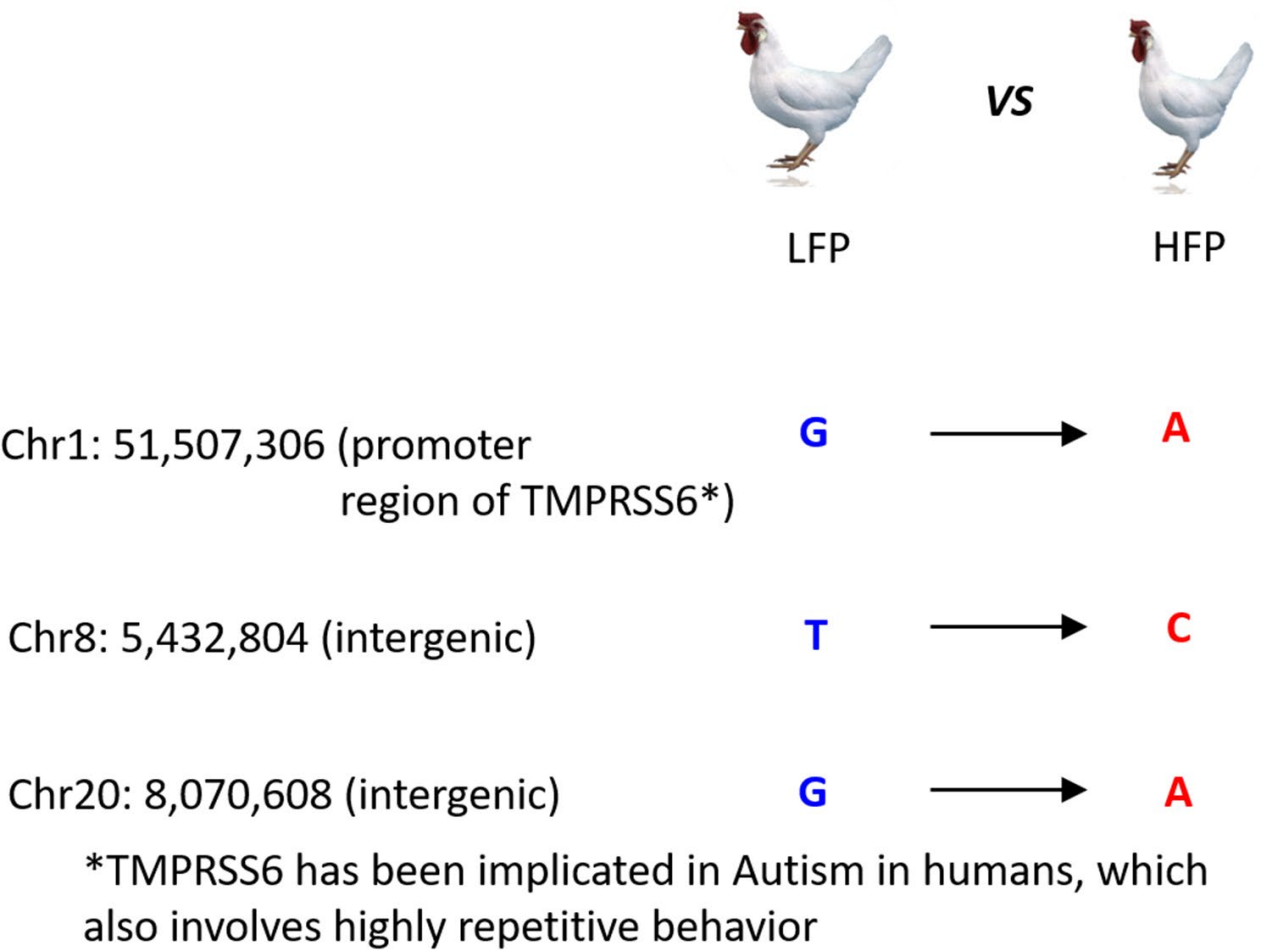
Schematic representation of the three loci showing differentially fixed alleles between HFP and LFP animals

We found three loci with differentially fixed alleles between HFP and LFP animals (Fig. 4). These were located in Chr1: 51,507,306 (G **>** A; promoter region of the *TMPRSS6* gene), Chr8: 5,432,804 (T > C; intergenic) and Chr20: 8,070,608 (G > A; intergenic). We then analysed SNPs found in exon regions (considering galGal6; coordinates converted using the LiftOver tool from GenomeBrowser) to investigate potential translational consequences of the emergence of SNPs. Three of these SNPs were classified as missense variants (chr8:6,369,137 T/C; chr9:15,869,069 A/G; chr15:6,383,633 A/G) in the transcripts of the *QSOX1*, *RNPEPL1* and *CUX2* genes, respectively, with moderate impact to the translated protein (Table 1). Because these allelic changes occurred in LFP animals (compared to the reference genome), translational effects derived from these missense mutations are expected in LFP but not in HFP animals. Interestingly, two of these missense SNPs were located in the G of CpG dinucleotides: an A/G substitution in the gene *RNPEPL1* (chr9:15,869,069) and an A/G substitution in the gene *CUX2* (chr15:6,383,633).

**Table 1:** Genes affected by Single Nucleotide Polymorphisms obtained in exonic regions between HFP and LFP, based on the chicken reference genome (GGA6). Information on gene function obtained from Uniprot (https://www.uniprot.org/) and QuickGO (https://www.ebi.ac.uk/QuickGO/)

Gene abbreviation	Gene Name	Involved in…	Part of…	Enables…	Effect in…
CUX2	Homeopbox protein cut-like	Positive regulation of synapse assembly; cellular responses to organic substances; Golgi vesicle transport; regulation of transcription; positive regulation of dendritic spine morphogenesis, excitatory postsynaptic potential, and synapse assembly; short-term memory (cognition)	Nucleus; Golgi membrane;	RNA polymerase II regulatory region sequence-specific DNA binding; transcription repressor activity	LFP (via G)
QSOX1	Sulfhydryl oxidase 1	Catalysing the oxidation of sulfhydryl groups in peptide and protein thiols to disulphides; disulphide bond formation in a variety of extracellular proteins; incorporation of laminin into the extracellular matrix in fibroblasts, affecting cell-cell adhesion and cell migration; cell redox homeostasis; negative regulation of macroautophagy; extracellular matrix assembly; protein folding	Golgi membrane; extracellular space and exosomes; intercellular bridge; intracellular membrane-bounded organelle	Flavin-linked sulfhydryl oxidase activity; protein disulphide isomerase activity; FAD binding; thiol oxidase activity	LFP (via C)
RNPEPL1	Leuk-A4-hydro_C domain-containing protein	Proteolysis	Nucleoplasm and nuclear bodies	Metalloaminopeptidase activity; zinc ion binding	LFP (via G)

We also investigated if, across the lineages, SNPs would occur more often in the C position of CpG dinucleotides (CpG-SNPs) defined by the reference genome. This would imply CpG disappearance and thus elimination of the possibility of DNA methylation in that C. As expected, the emergence of CpG-SNPs was above expectancy in general, with an occurrence of 1.4X above expectancy, and specifically for promoters (1.77X above expectancy) and introns (1.52X above expectancy) (*P* < 0.01, see Table 2). We found 62 CpG-SNPs among the 711 significant SNPs, representing 8.7%. We found that in 48.3% of these CpG-SNPs the C is maintained as the most frequent base in HFP, while the C is the most frequent base in 56.9% of cases in LFP (see Additional file 3 Table S2). Most of the changes observed between the reference genome and HFP or LFP have been C→T, with a few C→A and C→G also occurring. CpG-SNPs occurred mostly in introns, followed by distal intergenic regions, and promoters (Fig. 3b). However, they are significantly above expectancy only in promoters and introns (Table 2).

**Table 2:** Functional genomic annotation of SNP CpG-SNP emerging between high and low FP chicken selection lines. In bold, the significant differences in relation to expectancy

	SNPs found	CpG-SNPs expected (1/16 of SNPs found)	CpG-SNPs found	Fold change of CpG-SNPs in relation to expectancy	Chi Square result
**Exon**	14	0.9	0	0.00	Non-significant
**Intron**	253	15.8	24	1.52	*P* < 0.05
**3’ UTR**	6	0.4	0	0.00	Non-significant
**Promoter**	153	9.6	17	1.77	*P* < 0.05
**Downstream**	17	1.1	0	0.00	Non-significant
**Distal Intergenic**	268	16.8	21	1.25	Non-significant
**Total**	711	44.4	62	1.40	*P* < 0.01

We also investigated whether the SNPs found were involved in the emergence of novel CpGs in any of the selection lines. For this, we selected all the C-containing SNPs that neighboured a G in their 3′ end, but that didn’t contain a C in the reference genome; these were considered as ‘novel CpGs’. We found 56 novel CpGs that emerged in the lineages. Of these, 25 CpGs had C as the most frequent allele in HFP animals, while 31 CpGs had C as the most frequent allele in LFP animals (see Additional file 4 Table S3). Novel CpGs emerged in different genomic regions compared to the disappearance of CpGs (CpG-SNPs) (Fig. 3b and c). The appearance of novel CpGs took place mostly in distal intergenic regions, followed by introns and promoters, while CpG-SNPs occurred mostly in introns. However, for neither CpG-SNPs or novel CpGs the frequency is significantly different from that of all the SNPs (Chi-Square). The complete information related to CpG loss or appearance is available in Additional file 5 Table S4.

### CNV calling

**Fig. 5:**
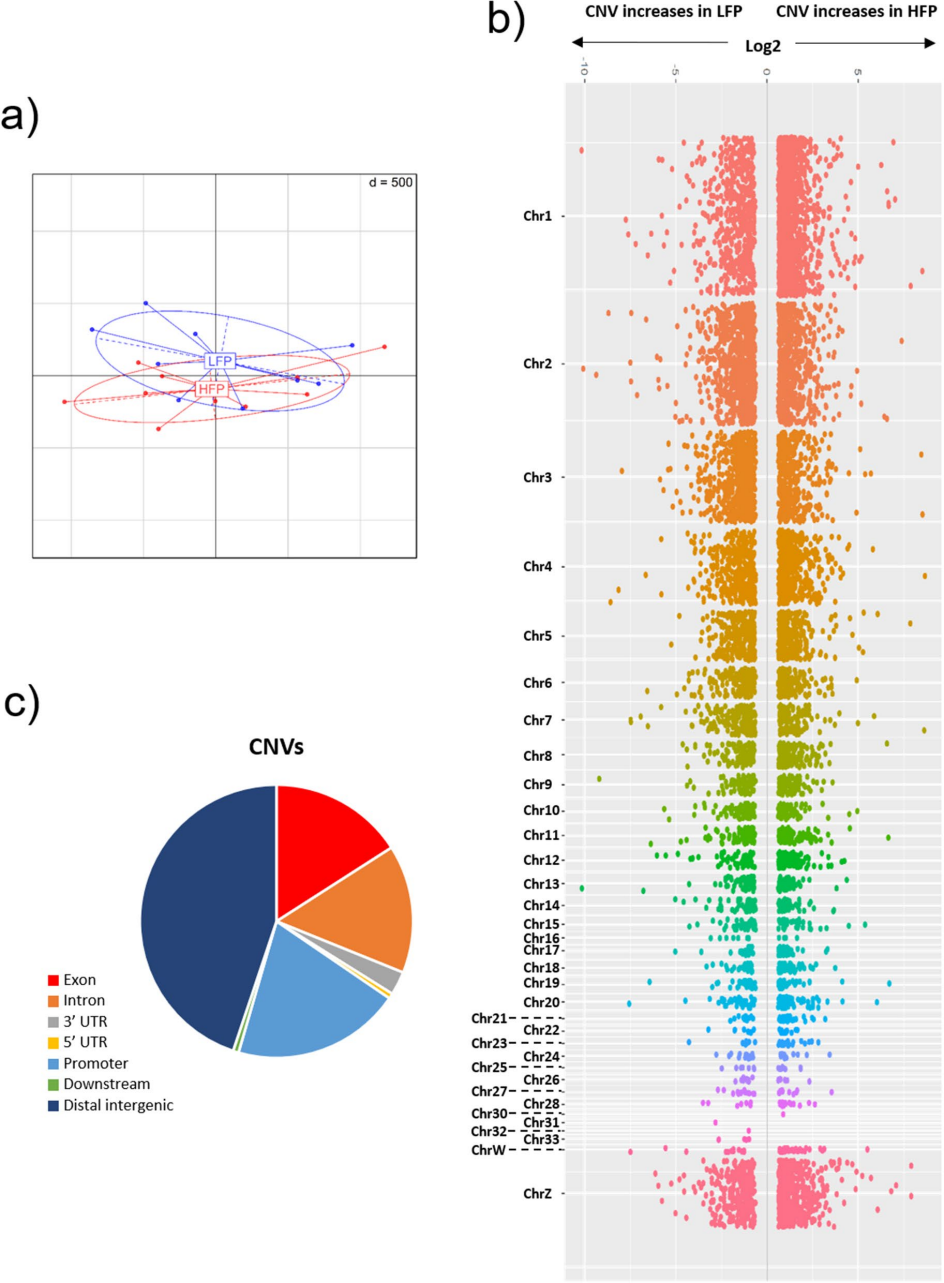
**a**) PCA showing genetic separation of individuals in each lineage (9 HFP and 7 LFP) based on CNVs; **b**) Manhattan plot showing CNV increases in each lineage across chromosomes; **c**) Functional annotation of CNVs found between LFP and HFP animals

The chicken genome size used for the CNV calculations was set at 1,050,947,331 bp. The window size used was 3,410 bps according to the CNV-seq package recommendations [59]. We compared 64.9 million reads from the HFP against 60.9 million reads from the LFP. The program estimated 10,128 CNVs (Program’s default Bonferroni, P-value < 4.668925 × 10^− 7^) with an average size of 7914 bps (median of 17,049 bps) encompassing 83,284,125 bps, which corresponds to 8.2% of the chicken genome (see Additional file 6 Table S5). A PCA based on the log2-normalized read counts of the CNVs found (Fig. 5a) shows a slight separation between HFP and LFP, smaller than the one observed for SNPs. Among all the omic levels tested, CNVs were the only ones yielding significantly enriched pathways at Padj < 0.05 (see Additional file 7 Table S6). The genomic location of the majority of the CNVs was in distal intergenic regions (44.9%), followed by promoter (19.1%), exonic (15.9%), and intronic (15.2%) regions (Fig. 5b). HFP chickens presented 58% of copy number gains and 42% of copy number losses compared to the LFP line. Large fold changes, above 3X, representing gains in one of the groups are observed in most of the chromosomes (Fig. 5c). GO Enrichment analysis of this subset of gene-associated CNVs shows involvement in biological processes related to nervous system development, such as neuron projection/guidance, chemotaxis, and synaptic assembly (Table 3). We considered as top CNVs those with a minimum of 8X fold-change in one group relative to the other (see Additional file 8 Table S7). The top CNV gains identified in HFP were located in promoter regions of the genes *ARSJ*, *PUM2* and *MTRF1* and in intronic regions of the gene *LOC771456*. In turn, the top CNV gains identified in LFP were located in promoter regions of the gene *SENP2*, in intronic regions of the gene *GABBR2*, in exonic regions of the gene *TRPC7*, in the 3’ UTR of the gene *SEPSECS*, and in the 5’ UTR of the gene *PTPRA*. The largest CNV associated to a gain in HFP involves 23,869 bp and associates with promoter regions of the *RIC3* gene, while the largest CNV associated to a gain in LFP involves 22,165 bp and associates with promoter regions of the *SH3RF1* gene.

**Table 3:** GO Biological Process pathways enriched by genes associated to CNVs with fold change over 3X

	Source	Term name	Term ID	Adjusted *p* value
**CNVs > 3x HFP**	GO: BP	axon guidance	GO:0007411	0.011
	GO: BP	Chemotaxis	GO:0006935	0.001
	GO: BP	multicellular organism development	GO:0007275	0.006
	GO: BP	neuron projection guidance	GO:0097485	0.012
	GO: BP	regulation of fibroblast growth factor receptor signaling pathway	GO:0040036	0.005
	GO: BP	system development	GO:0048731	0.015
	GO: BP	Taxis	GO:0042330	0.001
**CNVs > 3x LFP**	GO: BP	cell junction assembly	GO:0034329	0.005
	GO: BP	ureter development	GO:0072189	0.005
	GO: BP	positive regulation of synapse assembly	GO:0051965	0.006
	GO: BP	cell-cell adhesion	GO:0098609	0.007
	GO: BP	synapse assembly	GO:0007416	0.008
	GO: BP	positive regulation of multicellular organismal process	GO:0051240	0.009
	GO: BP	regulation of synaptic transmission, glutamatergic	GO:0051966	0.017
	GO: BP	cell adhesion	GO:0007155	0.018
	GO: BP	regulation of synapse assembly	GO:0051963	0.019
	GO: BP	regulation of nervous system development	GO:0051960	0.025
	GO: BP	positive regulation of nervous system development	GO:0051962	0.031

### DMR calling

**Fig. 6:**
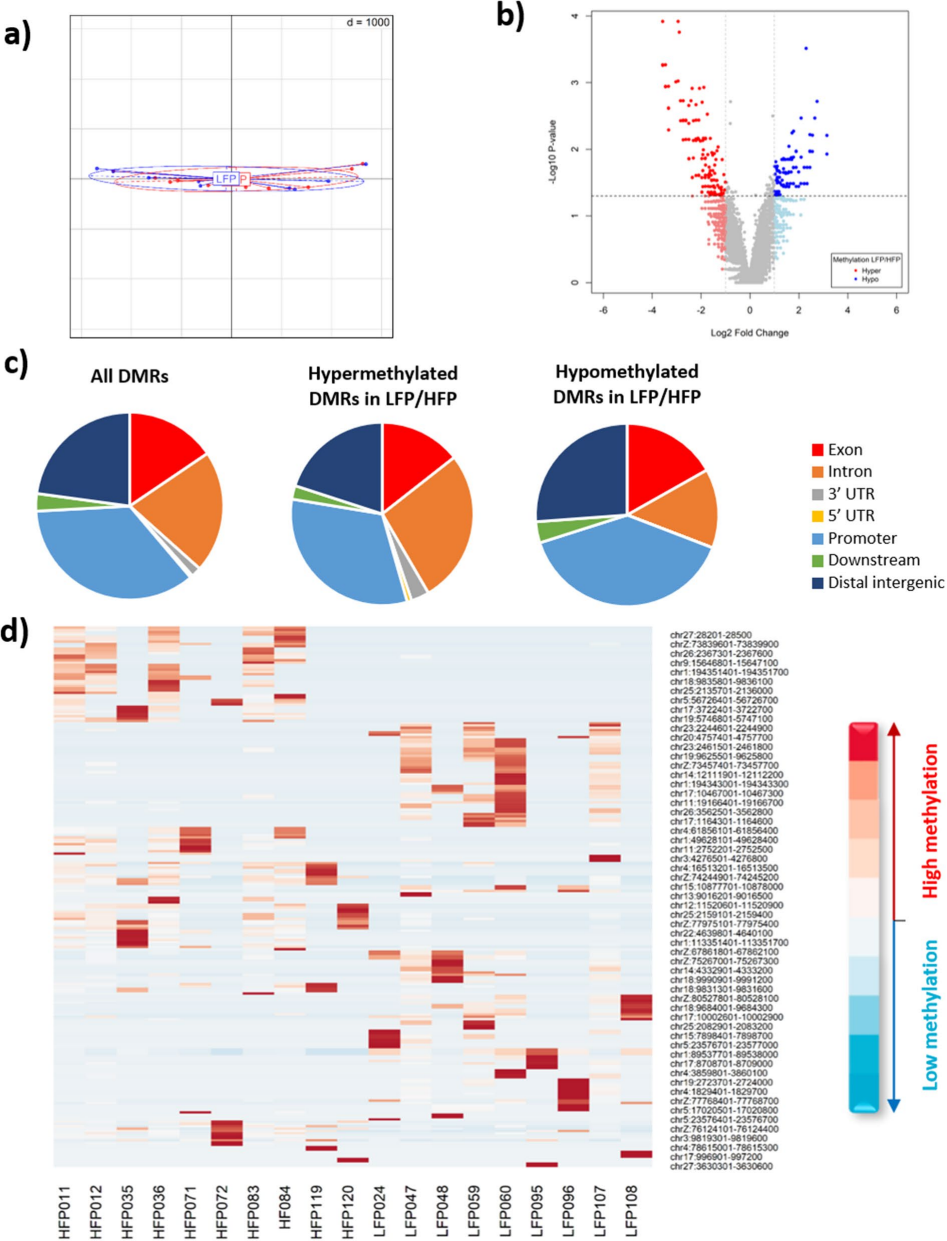
**a**) PCA showing methylomic separation of individuals in each lineage (9 HFP and 7 LFP) based on DMRs; **b**) Volcano plot showing fold changes of DMRs found between LFP and HFP animals; **c**) Functional annotation of DMRs found between LFP and HFP animals; **d**) Heat map showing methylation levels of the significant DMRs per individual

We covered 0.6 ± 0.2% of the genome using GBS-MeDIP. We obtained CpG enrichment scores of 1.81 for the GBS and 3.27 for the GBS-MeDIP compared to a base score of 1.12 from the Chicken Reference Genome (BSgenome.Ggallus.UCSC.galGal5). From a total of 3,396,079 windows of 300 bps from the chicken genome that could be analysed for the DNA methylation in the thalamus, 8910 were considered to be differentially methylated regions (DMR) between LFP and HFP animals. These 8910 windows passed a default minimum row sum threshold (minRowSum = 10) for reads counted for 9 LFP and 10 HFP chickens. From these windows, 232 were considered significant DMRs (P-value ≤ 0.05) between the HFP and LFP lines. A PCA based on the log2-normalized read counts of the DMRs found (Fig. 6a) shows no separation between HFP and LFP, contrasting with the observations for the SNPs and CNVs. Of the 232 significant DMRs, 107 DMRs (46.1%) were hypomethylated in LFP in relation to HFP, while 125 DMRs (54.9%) were hypermethylated in LFP in relation to HFP (Fig. 6b). Most of the DMRs were located in promoter (35.3%) followed by distal intergenic (22.8%), intronic (21.1%) and exonic (15.5%) regions (Fig. 6c). Figure 6d shows methylation levels of the significant DMRs per individual investigated. We then investigated separately the genomic locations of all the DMRs, as well as separated by being hypo- or hypermethylated in LFP compared to HFP (Fig. 6d). The genomic locations of the hypo- or hypermethylated DMRs in LFP compared to HFP were significantly different. An important difference is that hypomethylated DMRs in LFP are nearly halved in intronic regions compared to the hypermethylated in LFP (Fig. 6d). Also, hypermethylated DMRs in LFP were observed in 5′ and 3′ UTR, contrasting with the lack of these regions for the hypomethylated DMRs in LFP. The full list of significant DMRs and their genomic location is provided in Additional file 9 Table S8.

To investigate the relationship between DMRs and genes, we built a list of merged adjacent DMRs associated to genes (see Additional file 10 Table S9). In total, we found 108 genes associated to 166 merged DMRs, henceforth named gene-related DMRs (GR-DMRs), and 8 genes contained DMRs that passed the more stringent P-value of *P* ≤ 0.005 (Table 4). Functional enrichment analysis of these 108 GR-DMRs (performed by the g: Profiler web based tool, https://biit.cs.ut.ee/gprofiler/gost) revealed enrichment of 101 transcription factor (TF) binding sites (*P* ≤ 0.05; see Additional file 11 Table S10). Since hyper- or hypo-methylated DMRs might differ in their molecular action, we performed a g: Profiler functional enrichment analyses separately on the hyper- and hypo-methylated GR-DMRs in LFP relative to HFP. While no TF binding sites was found enriched in the 58 GR-DMR hypomethylated in LFP, 15 TF binding sites were enriched in the 53 GR-DMR hypermethylated in LFP (FDR *P* ≤ 0.05) (see Additional file 12 Fig. S2). Of special relevance, 3 genes (DCHS1, RBFOX3, SLC12A5) contained more than one DMR and these displayed opposite directional changes in DNA methylation (highlighted in yellow in Additional file 11 Table S10).

**Table 4:**
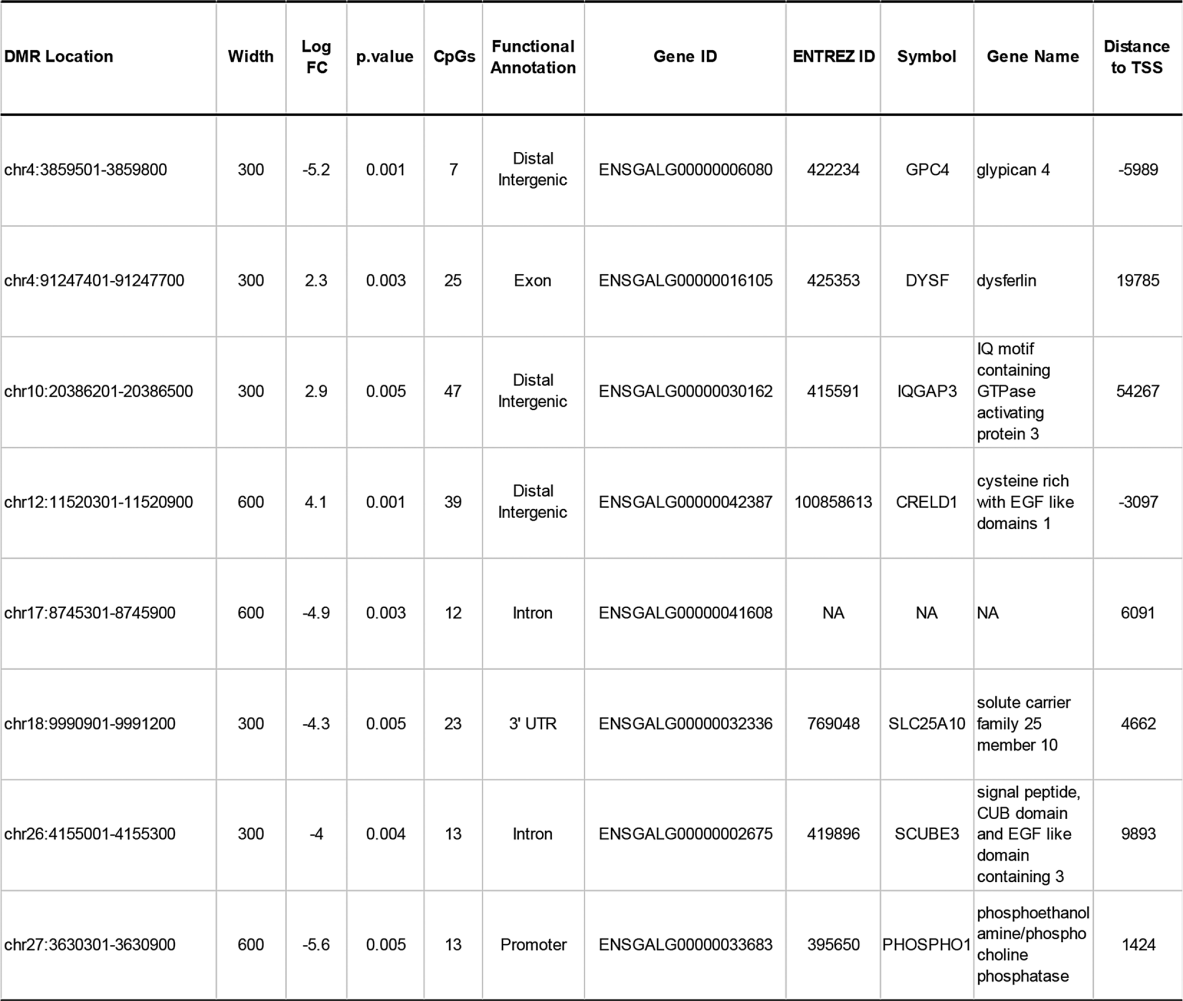
Descriptive statistics and gene annotations of genomic regions in the thalamus differentially (*P* ≤ 0.005) methylated (DMRs) between HFP and LFP lines

### Comparison with QTLs

**Fig. 7:**
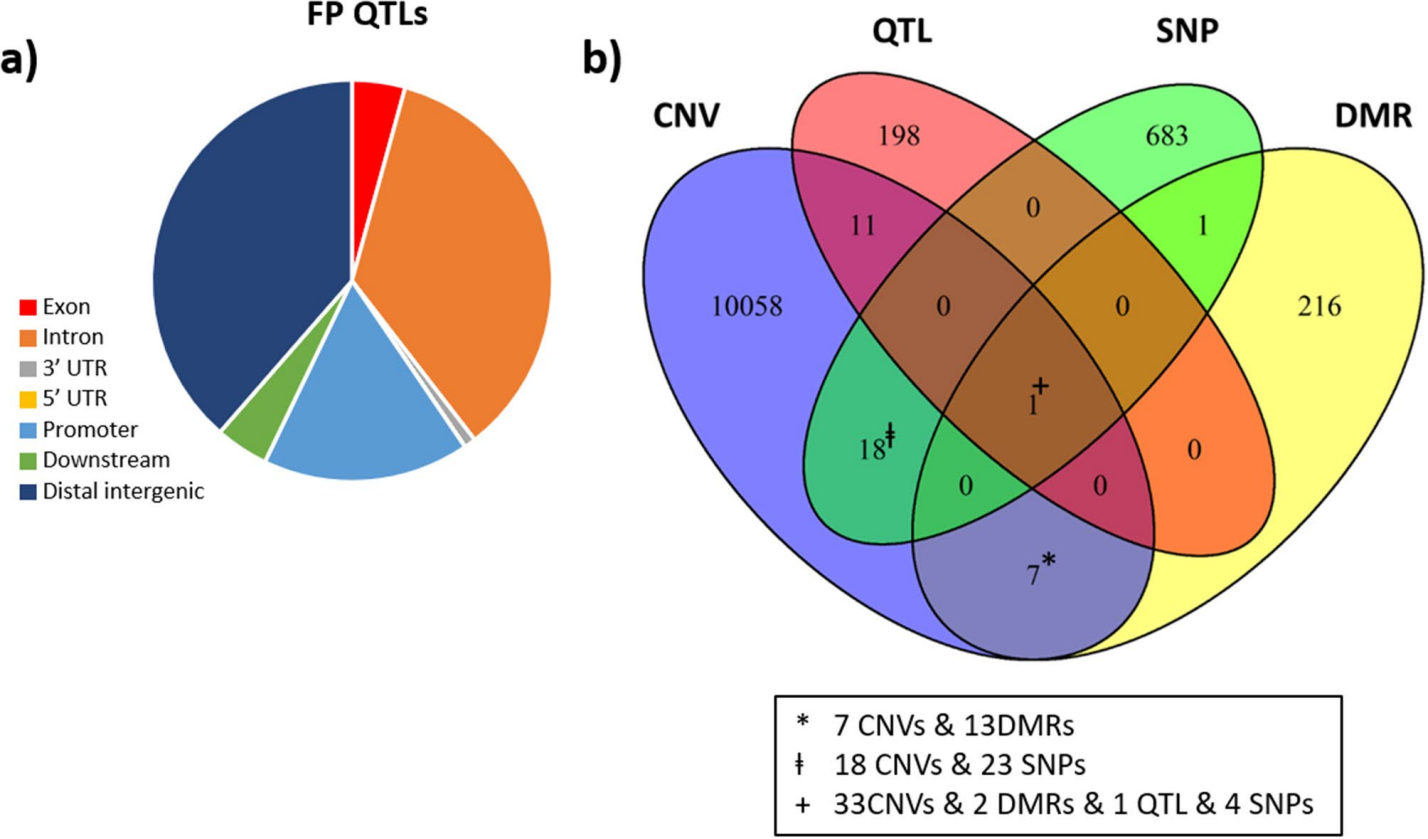
**a**) Functional annotation of publicly available QTLs for feather pecking behaviour; **b**) Venn diagram depicting the total number of genetic and epigenetic difference between animals in estimated in this study, as well as the common among them and publicly available QTLs for feather pecking

We also investigated how the different genomic levels assessed in this study (i.e., SNPs, CNVs, DMRs) compared among them and to previously annotated QTLs for performing and receiving ‘FP’ (https://www.animalgenome.org/cgi-bin/QTLdb/GG/index). We observed that QTLs (Fig. 7a) and SNPs (Fig. 3a) were very similar in their genomic locations, with very little presence in exons and high presence in introns. CNVs, in turn, were less present in introns and highly present in distal intergenic regions (Fig. 5c), while DMRs were less present in distal intergenic regions and highly present in promoter regions (Fig. 6d).

### Overlaps between the omic levels investigated

**Fig. 8:**
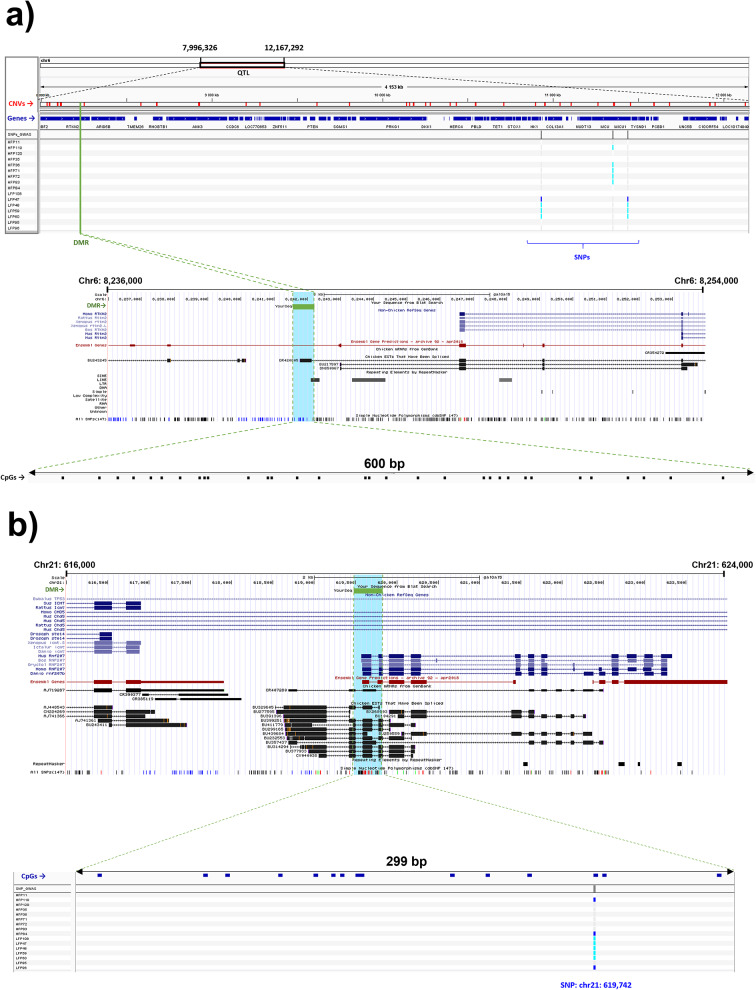
Graphical representation of overlaps of interest: **a**) the only region overlapping all the genomic levels investigated, which included a QTL for feather pecking, 33 CNVs, four SNPs and two adjacent DMRs (merged into one in the figure), located in the promoter of *RTKN2*; **b**) a DMR containing 21 CpGs that overlapped with one SNP, located in the promoter region of the novel gene *ENSGALG00000032525*

We then investigated genomic location overlaps among the omic sets obtained in this study (i.e., the 711 SNPs, the 232 DMRs, and the 10,128 CNVs) and the 210 FP-QTLs. We found 38 overlaps among the omic levels investigated (Fig. 7b, see Additional file 13 Table S11), with one overlap including all the omic levels. This extensive overlapping region locates within a previously mapped QTL (chr6: 7,996,326 − 12,167,292; PUBMED ID: 28,158,968) for FP (www.animalgenome.org), which overlaps with 33 CNVs, four SNPs and two adjacent DMRs (Fig. 8a; see Additional file 13 Table S11). These four SNPs were the only ones overlapping with a known QTL for FP. The two DMRs, which are adjacent, contain 32 CpGs altogether and are located in the promoter of the gene *RTKN2*. Interestingly, one of the overlapping SNP (chr6:10,931,835) occurred in an intergenic region downstream of the same gene. In contrast to HFP individuals, LFP individuals presented the most frequent allele of this SNP, i.e., G, which is the alternative allele to the reference genome (see Additional file 2 Table S1).

Another overlap of interest was a DMR containing 21 CpGs that overlapped with one SNP (chr21:619,742 T > C; see Additional file 13 Table S11). This overlap occurred in the promoter region of a novel gene (*ENSGALG00000032525*; Fig. 8b). The overlapping SNP occurred in a CpG dinucleotide in the LFP line. Interestingly, in the HFP line this CpG is lost, which is concordant with the hypomethylation of the overlapping DMR observed in the HPF line (-logFC=-3.3) because of the loss of a methylatable site. Additionally, we found 19 overlaps between CNVs and SNPs, 12 overlaps between CNVs and QTLs, and 7 overlaps between DMRs and CNVs (Fig. 7b; see Additional file 13 Table S11). The identification of CNVs encompassing DMRs is important in order to detect CNVs that could be confounded as DMRs. However, out of the 232 significant DMRs found, only 13 could be confounded with CNVs (Fig. 7b).

### Repeat element analyses

**Fig. 9:**
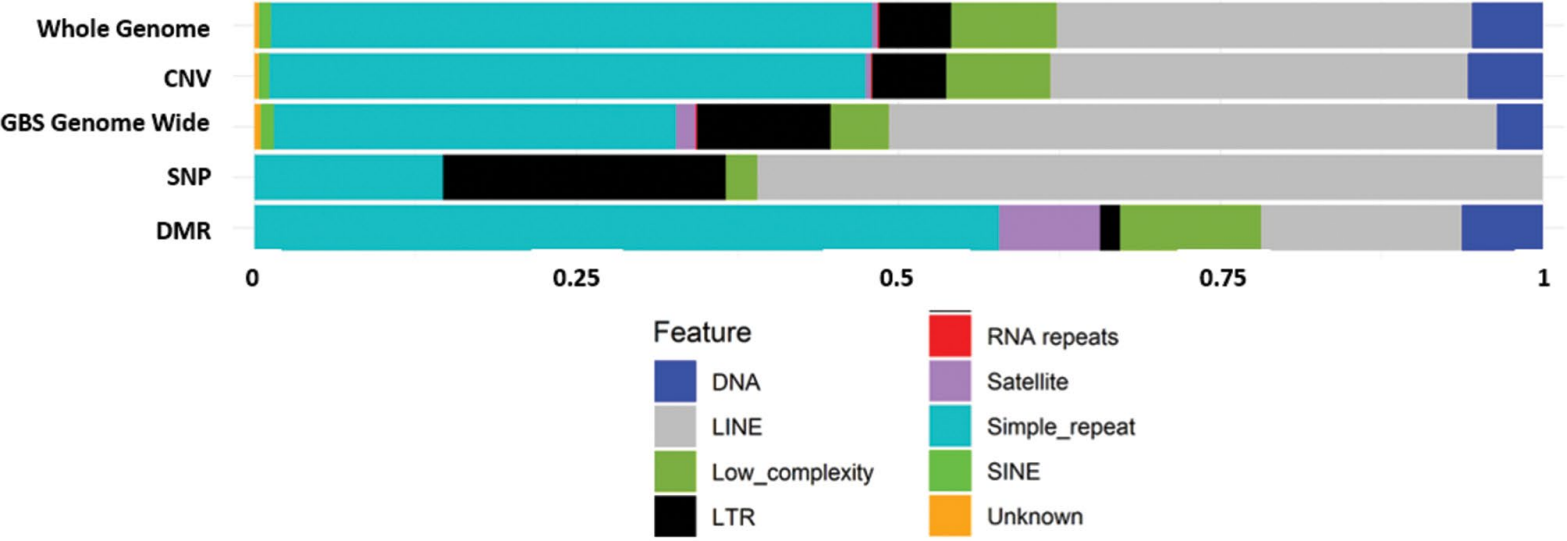
Graphical representation of repeat masker analysis on the SNPs, CNVs and DMRs found between LFP and HFP animals

We then performed repeat masker analysis on the SNPs, CNVs and DMRs obtained (Fig. 9), because of the relevance of repeat elements for the emergence of CNVs. We found that the number of repeats in each repeat element category is essentially the same for the CNVs and the whole chicken genome. However, it needs to be considered that our universe is the GBS fraction of the genome; when compared to that, the CNVs identified here associate with higher levels of simple repeats and lower levels of line elements. Surprisingly, however, DMRs are the ones that associate the most with simple repeats and the least with LTR and LINE elements. On the contrary, SNPs are the ones that associate the least with simple repeats and the most with LINE and LTR elements.

## Discussion

In this study, we compared genetic and epigenetic differences emerging in two chicken lines after being divergently selected for FP behaviour for seven generations and then maintained separately until the 18th generation. The analysis of this genetic and methylomic differences help to understand the effects selection has on genomes, as well as the molecular basis of FP behaviour. However, while our study provides valuable insights, an important limitation is the small sample size, which may affect the robustness, as smaller samples can increase variability and limit statistical power. This highlights the need for larger follow-up studies based on our findings.

Based on the 76,414 SNPs that passed the call rates, we analysed the separation of the individuals into groups by NJ, PCA, Fst, and Tajima D analyses. These analyses show that the two groups have achieved a discernible and quantifiable genetic separation in only seven generations of selection, which has resulted in population structuring, where only a few individuals are visualized in the intersection zone between the two groups. Concordant with previous findings, this selection against FP did not fully eliminate the occurrence of FP [21] in the LFP line. This is expected, as genomic variability is shown to remain in divergent populations when selection is on the extremes [21], as it is the case for our selection for FP.

Many of these SNPs showing strong signals of genetic differentiation between the lineages are located within or near genes involved in neurodevelopment, stress response, and cellular signalling, biological functions that are expected to be involved in FP. Importantly, we found two genes directly connected, functionally. One is *SST*, which encodes somatostatin, a neuropeptide involved in neurotransmission and behaviour regulation [65, 66]. The other is *ARNT2*, involved in neuronal development and axonal health [67], whose expression in somatostatin-expressing neurons within the prefrontal cortex is associated with affective state discrimination in relation to emotion recognition [68]. This suggests that genes involved in emotional and behavioural regulation may underlie FP and other repetitive behaviours. We found three loci with alleles differentially fixed between HFP and LFP animals (Fig. 4): a G **>** A substitution in the promoter region of the *TMPRSS6* gene (Chr1: 51,507,306), a T > C intergenic substitution (Chr8: 5,432,804), and a G > A intergenic substitution (Chr20: 8,070,608). The *TMPRSS6* gene transcribes for the transmembrane protease serine 6, involved in iron homeostasis [68], and is associated with iron imbalance in humans [69, 70]. Importantly, this gene has recently been singled out as a candidate for autism spectrum disorders based on evidence in humans, and by using exome sequencing and knockout in mice [70]. Because autism spectrum disorders in humans involve repetitive behaviour [71, 72], the finding of this fixed allele in the *TMPRSS6* gene of chickens in relation to FP (also a repetitive behaviour) may be of relevance to investigate the etiology of autism. The fixed or positively selected SNPs in these pathways indicate that selection has driven genetic divergence in loci influencing behaviour. Our findings point to key genomic regions for further investigation, and offer insights into the genetic architecture of FP behaviour.

Of the 711 SNPs found to be affected by the divergent selection on FP, only one (chr6:10931835 G > A) has been previously described to associate with FP [34]. Therefore, 710 SNPs are newly described here to associate with FP. Most of the SNPs are intergenic and intronic, followed by those in promoters. Of the significant SNPs identified in exonic regions, three are missense with moderate transcriptional impact in their encoded protein. These SNPs were observed in the exons of the genes *CUX2*,* QSOX1* and *RNPEPL1* and emerged in the HFP. Interestingly, the missense SNPs found in the exons of *QSOX1 and RNPEPL1* were in CpG sites, which suggests that they might have emerged secondary to a methylation gain in these CpGs in the germ line of their ancestors. These three genes relate to neuronal development and differentiation pathways. *CUX2* is involved in neurogenesis, specifically in the positive regulation of dendritic spine morphogenesis, excitatory postsynaptic potential, gene expression, and synapse assembly (UniProtKB - O14529). In the murine cortex, *CUX2* expresses in the pyramidal neurons of the upper layers (II–IV), and together with *CUX1* defines the identity of these neurons [73]. *CUX2*-expressing neurons are particularly vulnerable to damage induced by multiple sclerosis, showing upregulation of stress pathway genes [74]. Additionally, a region in the human genome that includes *CUX2* is shown to be implicated in the pathology of epilepsy [75]. The gene *QSOX1* (sulfhydryl oxidase 1) is involved in cell redox homeostasis, extracellular matrix assembly, negative regulation of macro autophagy and apoptosis (UniProt – Q8JGM4), which are mechanisms especially important in cellular responses. An extensive bioinformatic analysis using data from The Cancer Genome Atlas project revealed that *QSOX1* is one of the 20 genes shown to be overexpressed in glioblastoma multiforme, and one of the 8 genes associated with reduced survivability of patients carrying this disease [76]. *RNPEPL1*, in turn, is a rarely studied aminopeptidase with ubiquitous tissue expression, which is concordant with a housekeeping function [77]. Alternative splicing of its mRNA was observed in all tissues examined [77]. Aminopeptidases are involved in protein turnover and in the central nervous system mediate a variety of specialized functions. Inhibition of cerebral aminopeptidases is shown to induce analgesia, apoptosis, and amnesia [78]. The effects these three SNPs might have on protein structure and function in neural tissues remain to be investigated.

The significant SNPs are present 1.4X above expectancy within CpG nucleotides. These CpG-SNPs were found in similar amounts in HFP and LFP chickens. The presence of CpG-SNPs above expectancy in relation to genomic diversification is concordant with our previous findings in chickens [79]. Of further interest, the 7-generation long selection modified the chicken genome by favouring the emergence of SNPs in CpGs located in introns, distal intergenic regions, and promoters. Thus, these genomic regions seem to be hotspots for CpG depletion, which would imply the loss of the possibility of gene expression regulation by DNA methylation. CpG depletion is a commonly observed phenomenon in vertebrate genomes [56]. However, a significantly higher than expected CpG-SNPs occurrence was observed only in promoters (1.77X above expectancy) and introns (1.52X above expectancy), showing that the hypermutability of CpG sites is not affecting genomic regions equally. Because CpG depletion has been associated to the emergence of transcription factor binding sites (TFBS) during evolution [80], our data on CpG depletion suggests that promoters and introns could be hotspots of TFBS origination during selection processes. Additionally, we investigated whether the SNPs found were involved in the emergence of novel CpGs in each selection line. Novel CpGs also appeared to a similar extent in both selection lines. The consequence of the emergence of novel CpGs is the acquisition of an epigenetic level of regulation that was not present before. This represents a new ability of regions such as distal intergenic regions, introns, and promoters to be methylated.

One of the most striking observations is the number of CNVs that emerged between the selection lines compared to the SNPs and DMRs. CNVs are reported to have important consequences for genomic evolution and involve genomic rearrangements such as duplications, triplications, inversions and other that can fuse or disrupt genes, or produce tandem repeats [81]. CNVs have been described as a source of genetic diversity in the evolution of well-diverged species such as chimpanzees and humans [82], and in more recent diversification events such as between wolves and dogs [83], and wild boars and pigs [84]. To the best of our knowledge, this is the first study that has investigated the emergence of CNVs within the context of a controlled process of artificial selection. CNVs created by the FP artificial selection affected nearly 8% of the chicken genome (83,234,125 bp affected) compared to 711 SNPs and 232 DMRs (300 bps*232 = 69,600 bps affected) found to be significant. This points to the relevance of the emergence of CNVs during selection compared to SNPs and methylomic changes. The importance of CNVs for bird evolution is documented in great tits, where CNV breakpoints (which are CpG rich) are observed in nearly half of their genes, locating prominently at repetitive (segmental duplications) and regulatory regions, overlapping with transcription start sites [85]. CNVs generally appear in the germ line as the result of the activation of transposons that are de-repressed by epigenetic mechanism [86, 87]. Interestingly, the copy number gains (58%) observed here are more numerous than the losses (42%) in HFP chickens compared to LFP. This indicates that the selection applied is biased towards genomic events such as duplications in the HFP line, while biased towards deletions in the LFP line. The functional genomic consequences of this are unknown. CNVs were mainly located in distal intergenic regions, followed to a lesser extent in promoters, exons, and introns. Large CNVs can encompass one or many genes, as it is observed in great tits, where a CNV of approximately 2.8 Mb harbours the downstream breakpoint of a low frequency but large inversion that encompasses most of Chr1A (approximately 1000 genes) [88]. In our case, the largest gene-related CNV gain found in each lineage were associated to *RIC3* (promoter) in HFP and to *SH3RF1* (promoter) in LFP. *RIC3* is involved in the regulation of the expression of nicotinic acetylcholine receptors, both via *RIC3* expression and splicing [89] *RIC3* is a putative locus involved in promoting healthy cognitive aging [90]. Additionally, a *RIC3* variant has been shown to associate with backward speech in humans [91]. Of special interest is the finding of CNV gains in LFP in intronic regions of *GABBR2*. This gene belongs to the GABA-B subfamily of G-protein coupled receptor 3 family. Altered GABA-B receptor function relates to a variety of neurological and psychiatric disorders, including epilepsy, depression, drug addiction, cognition, and nociception [92]. Importantly, GABA receptors are factors associated with an increased propensity of FP in laying hens [38]. *GABBR2*, in particular, is a crucial factor in neurodevelopmental phenotypes, with mutations being associated with Rett syndrome and epileptic encephalopathy [93]. *SH3RF2*, in turn, is a recently described oncogene in humans [94], concordant with recent research showing the presence of CNVs being frequent across several cancer types [95].

The substantial fraction of the genome affected by CNVs in this study after only 7 generations of selection suggests that CNVs are an important and underestimated initial step for the genomic diversification of species, possibly with larger implications than SNPs. The finding that the main biological function affected by CNVs is neuronal development (according to GO analysis), highlights the connection between this type of genomic alterations and the phenotypic differences between the two lineages investigated. We also compared the genes associated with the SNPs, DMRs and CNVs found here with genes previously found to have altered gene expression in chicken hypothalamus in relation to FP [36]. Only CNVs presented concordant genes. These CNVs exhibited small albeit significant fold changes and were distal intergenic in relation to *PLD5*, intronic in *MAPK8*, and exonic in *SRI*. *PLD5* has been recently described as pivotal in the brain development of children [96], while experiments in mice show *MAPK8* as one of the candidate “coordinator” genes involved in the pathogenesis of psychosomatic pathologies caused by chronic social stress [97]. *SRI*, in turn, is an early marker of neurodegeneration, acting via calcium signalling dysregulation [98]. The results of our GO analysis on CNV-related genes and the relation of our CNVs to genes with previously identified expression changes in the hypothalamus in FP points to the importance of CNVs in normal and pathological neurodevelopment.

In relation to epigenetic differences, 54.9% of the DMRs were hypermethylated in the LFP line compared to HFP (Fig. 6b). These epigenetic changes have accumulated over multiple generations, possibly as a consequence of the genomic changes produced by the selection process, because the two selection lines have been reared and maintained under the same environmental conditions. Similarly, in red jungle fowl chickens, gene expression and methylomic changes emerge in the hypothalamus after only 5 generations of divergent selection for high or low fear of humans [41]. The merged DMRs found here were associated with 108 genes. In general, DMRs occurred mostly in promoter regions (35.3%), followed by distal intergenic (22.8%) and intronic (21.1%) regions. However, changes to this pattern were observed when investigating the directional changes in methylation separately in each selection line. For example, hypomethylation in LFP (compared to HFP) animals occurred to an even higher level in promoters (39.3%) and distal intergenic regions (26.2%), while hypermethylation in LFP (compared to HFP) animals was higher in intronic regions (27.2%) compared to all the DMRs and hypomethylated in LFP. Additionally, hypomethylated DMRs in LFP (compared to HFP) animals are not observed in 5´and 3’ UTRs, while observed to a low level in LFP animals (~ 4% combined). These patterns point towards selection differentially acting on the epigenetic make up of specific genomic locations: while methylation tends to increase in response to selection in promoter and distal intergenic regions in HFP animals, it tends to increase in intronic regions and UTRs in LFP animals. We have found only one other study in the literature that investigated the differential effect of selection in the methylation status of functional genomic regions. This was performed in the plant *Brassica rapa* [99] and the authors show that CG and CHG methylation levels of positively selected genes are significantly higher in introns and UTRs compared to promoter and exon regions [99]. Interestingly, this is the same pattern observed in our LFP animals. Because the selection pressure is reported to be higher on HFP than LFP animals [47], this may indicate that relaxed selection on LFP animals would promote methylation in introns and UTRs. Conversely, higher selection pressures (e.g., negative selection) would cause increased methylation in promoter and exonic regions (as observed in our HFP animals), which are normally hypomethylated [100]. This hypothesis can be tested in other tissues and organisms. The genetic location where methylation changes occur is relevant for gene-expression regulation. As a general trend, hypermethylation in promotor regions is usually associated with gene repression, while hypermethylation in exon/introns (genic region) is associated with gene expression [101–103].

Interestingly, none of the 58 gene-related DMRs hypermethylated in HFP was found to be enriched for TFBS, while 28.3% of the 53 gene-related DMRs hypermethylated in LFP were enriched for TFBS. This means the 7-generation long selection for FP has led to sustained hypermethylation of genomic regions containing TFBS in the thalamus of LFP chickens. Independent of the mechanistic origin of these differences, there could be consequences for the epigenetic regulation of gene expression in these regions. Because DNA methylation is shown to repress TF binding [100], the binding of TF would be allowed in the hypomethylated regions in the thalamus of HFP chickens, while prevented in the hypermethylated regions in the thalamus of LFP chickens.

Three genes associated to the DMRs found are of particular interest because they contain more than one DMR and these display opposite methylation directions: *DCHS1*,* RBFOX3*,* SLC12A5*. These genes are new potential players to understand gene regulation in FP behaviour. Although *DCHS1* is an uncharacterized protein in chicken (www.uniprot.org), in other species this gene is involved in biological processes such as homophilic cell adhesion via plasma membrane adhesion molecules [104]. *RBFOX3*, in turn, is a RRM domain-containing protein important for nervous system development [105] and implicated in the regulation of alternative mRNA splicing via spliceosome [104]. *SLC12A5* (also known as RCC2) is an integral membrane K-Cl co-transporter, uncharacterized in chicken. In humans, *SLC12A5* is expressed exclusively in the brain, having a critical role in maintaining chloride homeostasis in neurons and being involved in fast post-synaptic inhibition [106]. Importantly, variants of this gene in individuals with autism have been associated with increased DNA methylation in its 3’ region [107]. Additionally, animals with reduced expression of this transporter exhibit severe motor deficits, epileptiform activity, and spasticity [108].

We also investigated whether the genomic regions affected by the FP selection in one assay overlapped with regions affected at other levels and with previously described FP QTLs. The main overlap found was a QTL (chr6: 7,996,326 − 12,167,292) that overlapped with many CNVs, three SNPs and one DMR (Fig. 8a). The DMR within this FP QTL (QTL_ID: 137239) [34] contained 32 CpGs, which were linked to the gene *RTKN2*, an oxysterol stress responder. In the brain, *RTKN2* participates in the downstream transcriptional regulation of the amyloid precursor protein, an important player in Alzheimer’s disease [109]. Interestingly, repetitive behaviours are a well-known symptom in Alzheimer’s disease [110]. Therefore, *RTKN2* could be an important gene in relation to the emergence of repetitive behaviours across vertebrates, which has not been investigated in this context. Another overlap of interest occurred between a SNP and a DMR containing 21 CpGs, located on a promotor region of the novel gene *ENSGALG00000032525* (Fig. 8b).

Finally, the repeat masker analyses performed across the different levels investigated revealed differences in their repeat element composition. DMRs are the ones that associated the most with simple repeats and the least with LTR and LINE elements, while SNPs are the ones that associate the least with simple repeats and the most with LTR and LINE elements. The fact that DMRs emerging during this selection process associate mainly with simple repeats suggests they may be a target of diversification via changes in DNA methylation. Simple repeats are tandem repetitions of short genomic motifs (1–6 bp) that have been implicated in genetic variation and genomic plasticity [111, 112]. In rats, simple repeats are the most methylated class of DNA/RNA repeats [113]. Interestingly, nearly half of the chicken genome represents simple repeats, meaning that it is quite enriched for simple sequence repeats compared to other organisms. While plant genomes are reported to contain less than 1% of single sequence repeats and fish and human genomes are reported to contain around 1–4% [112, 114], a value of 23% (e.g., in the genome of penaeid shrimp) is already considered of high simple sequence repeats content [111]. Simple sequence repeats are suggested to emerge from DNA polymerase slippage when one DNA strand temporarily dissociates from the other, and are often found in the proximity of interspersed repetitive elements such as short interspersed repeats (SINEs) and long interspersed elements (LINEs) [112].

SNPs emerging in the present selection process were highly associated with LINE elements. LINEs are long retro-transposable elements that encode all the enzymatic machinery needed for their transposable activity, and are able to mobilize nonautonomous retrotransposons, as well as messenger and noncoding RNAs, leading to the generation of pseudogenes [115]. Although not much is known about the role of SNPs in LINEs, tag SNPs have been identified for the majority of human LINE-1, and the produced insertions are suggested to respond to positive selection [116]. Both of these findings in humans are concordant with our findings in chickens. Future research must uncover the role that SNPs within LINE elements play in diversification following selection.

## Conclusion

Our study provides new knowledge on how selection affects genomic regions at different levels, namely, SNPs, CNVs and DNA methylation. The model investigated was artificial selection that produced two divergent lineages of chickens exhibiting high vs. low levels of FP behaviour. This is the first study that integrates data on DMRs, SNPs and CNVs during a controlled process of a vertebrate artificial selection to understand the underlying genomic and epigenomic dynamics. We identified 711 significant SNPs between the HFP and LFP individuals, out of which 710 are novel for FP. We found three loci having alleles differentially fixed between HFP and LFP animals. One of these is a G **>** A substitution in the promoter region of the *TMPRSS6* gene, implicated in autism (also a repetitive behaviour) in humans. Our Fst analysis revealed two important genes under strong selection that relate to somatostatin function, SST and *ARNT2*, and which could have an important role in FP via behavioural and emotional regulation. The significant SNPs found are present 1.4X above expectancy within CpG nucleotides. Because CpG-SNPs are above expectancy in promoters and introns, these genomic regions seem to be hotspots for CpG depletion, which would imply the loss of the possibility of gene expression regulation by DNA methylation. Compared to the other omic levels, CNVs exhibited the largest change during this artificial selection process. The selection applied is biased towards genomic events such as duplications in the HFP line, while biased towards deletions in the LFP line. Our findings in relation to genes related to CNVs points towards genes involved in regulation of nicotinic acetylcholine receptors (*RIC3*), GABA-B signalling (*GABBR2*), and oncogenesis (*SH3RF2*). Our study suggests that CNVs are an important and underestimated initial step for the genomic diversification of species, possibly with larger implications than SNPs. The CNVs found here are mainly involved in neuronal development, which is concordant with the phenotypic selection performed in the experiment and with previously reported gene expression data. When analysing the combined data from the different assays investigated, we found that the gene *RTKN2* (transcriptional regulator of the amyloid precursor protein) contain modifications in all the levels, and is, additionally, located in a QTL for the trait. These findings make *RTKN2* a very important candidate gene for future studies involving FP and other repetitive behaviours across vertebrates, especially considering its involvement in Alzheimer´s disease, where repetitive behaviours are an essential feature. *RTKN2* has not been investigated in the specific context of repetitive behaviours. The repeat analysis shows that the differences obtained in each assay performed here relate to different types of repeat elements, which may indicate specific responses of each of these genomic/epigenomic variants to selection, with concordant and different molecular effects and functions.

## Electronic Supplementary Material

Below is the link to the electronic supplementary material.

Supplementary Material 1: Additional File 1 Take ESM 1

Supplementary Material 2: Additional File 2 Take ESM 2

Supplementary Material 3: Additional File 3 Take ESM 3

Supplementary Material 4: Additional File 4 Take ESM 4

Supplementary Material 5: Additional File 5 Take ESM 5

Supplementary Material 6: Additional File 6 Take ESM 6

Supplementary Material 7: Additional File 7 Take ESM 7

Supplementary Material 8: Additional File 8 Take ESM 8

Supplementary Material 9: Additional File 9 Take ESM 9

Supplementary Material 10: Additional File 10 Take ESM 10

Supplementary Material 11: Additional File 11 Take ESM 11

Supplementary Material 12: Additional File 12 Take ESM 12

Supplementary Material 13: Additional File 13 Take ESM 13

## Data Availability

The data underlying this article are available in the European Nucleotide Archives (ENA) at the link www.ebi.ac.uk/ena/data/view/PRJEB35852, and can be accessed with the accession number PRJEB35852.
