# Correction: Genetic and neuro-epigenetic effects of divergent artificial selection for feather pecking behaviour in chickens

**DOI:** 10.1186/s12864-024-11200-6

**Published:** 2025-01-06

**Authors:** Elske N. de Haas, Fábio Pértille, Joergen B. Kjaer, Per Jensen, Carlos Guerrero-Bosagna

**Affiliations:** 1 Department of Veterinary Science, Animals in Science and Society, Utrecht University, Utrecht, The Netherlands; 2 Behavioural Ecology Group and Adaptation Physiology Group, Department of Animal Sciences, Wageningen University, Wageningen, The Netherlands; 3 Escola Superior de Agricultura “Luiz de Queiroz”, São Paulo, Brazil; 4 IFM Biology, Avian Behaviour Physiology and Genomics Group, Linköping University, Linköping, Sweden; 5 Federal Research Institute for Animal Health, Celle, Germany; 6 Department of Veterinary and Animal Sciences, University of Copenhagen, Copenhagen, Denmark; 7 Physiology and Environmental Toxicology Program, Department of Organismal Biology, Uppsala University, Uppsala, Sweden

**Correction: BMC Genomics 25, 1219 (2024)**

10.1186/s12864-024-11137-w

Following publication of the original article it was reported that the author affiliations for Elske N. de Haas, Fábio Pértille and Carlos Guerrero-Bosagna were assigned incorrectly.

The affiliations were wrongly given as:

Elske N. de Haas^1,2,7^, Fábio Pértille^3,4^, Carlos Guerrero-Bosagna^4^.

1 Department of Veterinary Science, Animals in Science and Society, Utrecht University, Utrecht, The Netherlands.

2 Behavioural Ecology Group and Adaptation Physiology Group, Department of Animal Sciences, Wageningen University, Wageningen, The Netherlands.

3 Escola Superior de Agricultura “Luiz de Queiroz”, São Paulo, Brazil.

4 IFM Biology, Avian Behaviour Physiology and Genomics Group, Linköping University, Linköping, Sweden.

The correct affiliations are:

Elske N. de Haas^1,2^, Fábio Pértille^3,7^, Carlos Guerrero-Bosagna^7^.

1 Department of Veterinary Science, Animals in Science and Society, Utrecht University, Utrecht, The Netherlands.

2 Behavioural Ecology Group and Adaptation Physiology Group, Department of Animal Sciences, Wageningen University, Wageningen, The Netherlands.

3 Escola Superior de Agricultura “Luiz de Queiroz”, São Paulo, Brazil.

7 Physiology and Environmental Toxicology Program, Department of Organismal Biology, Uppsala University, Uppsala, Sweden.

The correct full authorship is given in this Correction and the original article has been updated.

