# Supplementary figures and images for: Genetic and neuro-epigenetic effects of divergent artificial selection for feather pecking behaviour in chickens
Source: BMC Genomics. 2024 Dec 19;25:1219. doi: 10.1186/s12864-024-11137-w (PMC11657628; doi:10.1186/s12864-024-11137-w)

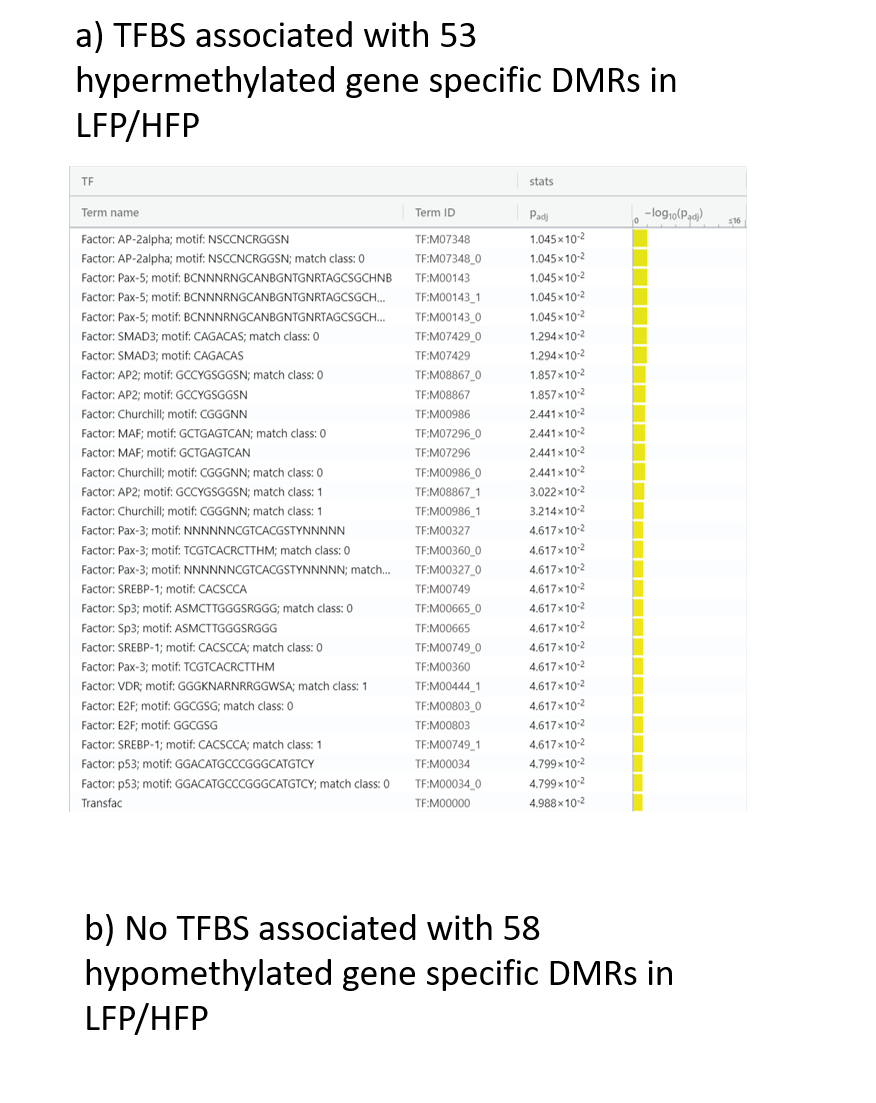

Supplement: Supplementary file 8 — Supplementary Material 8: Additional File 8 Take ESM 8 [file 12864_2024_11137_MOESM8_ESM.tif]
