# Supplementary material for: Genetic and neuro-epigenetic effects of divergent artificial selection for feather pecking behaviour in chickens
Source: BMC Genomics. 2024 Dec 19;25:1219. doi: 10.1186/s12864-024-11137-w (PMC11657628; doi:10.1186/s12864-024-11137-w)

Additional file 1

Fig S1 - Distribution of LD (r^2) values

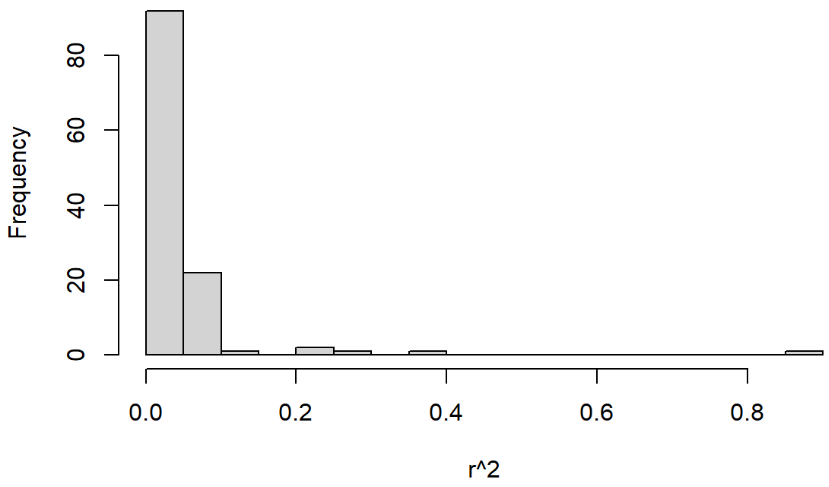

Fig S2 - Classification of SNPs obtained between LFP and HFP animals in relation to their Fst and Tajima's D values

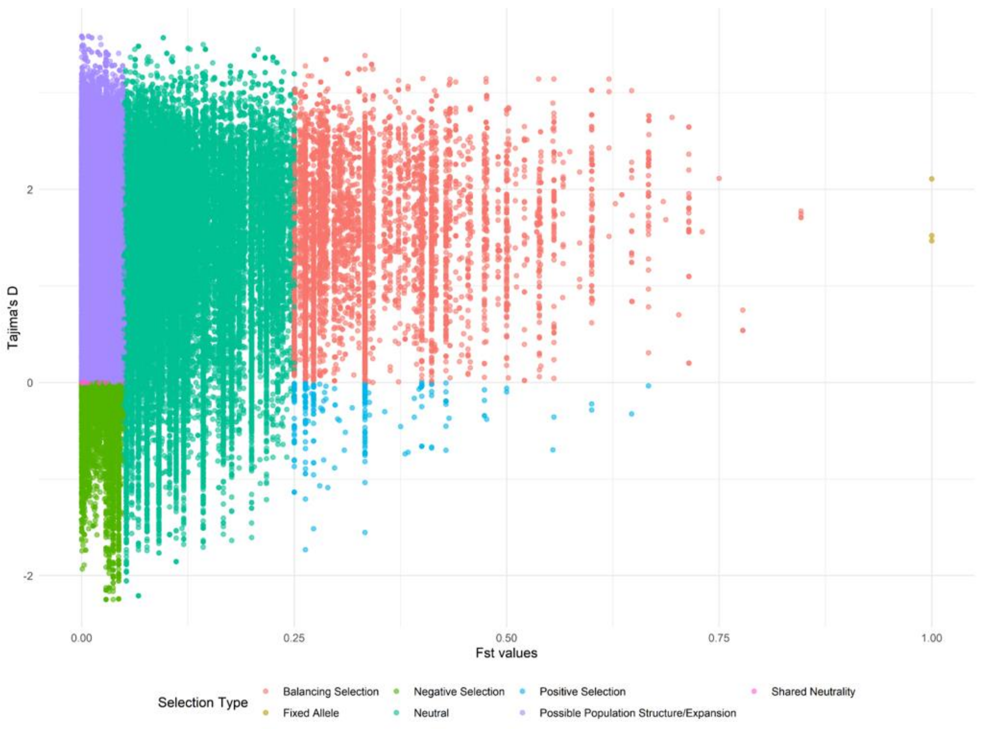

Supplement: Supplementary file 9 — Supplementary Material 9: Additional File 9 Take ESM 9 [file 12864_2024_11137_MOESM9_ESM.pdf]
