# Supplementary material for: Genetic and neuro-epigenetic effects of divergent artificial selection for feather pecking behaviour in chickens
Source: BMC Genomics. 2024 Dec 19;25:1219. doi: 10.1186/s12864-024-11137-w (PMC11657628; doi:10.1186/s12864-024-11137-w)
Supplement: Supplementary file 10 — Supplementary Material 10: Additional File 10 Take ESM 10 [file 12864_2024_11137_MOESM10_ESM.pdf]

Supplementary Table S2: Summary of CpG-SNPs between LFP and HFP animals

	SNP Position	Reference	HFP Most Frequent Allele	LFP Most Frequent Allele	Functional Annotation	Gene
**1**	chr1:166568	C	C	T	Intron	SHANK3
**2**	chr1:104617291	C	C	T	Distal Intergenic	SOD1
**3**	chr1:132017347	C	T	C	Distal Intergenic	CNGA3
**4**	chr1:169272049	C	C	T	Promoter	ARL11
**5**	chr1:186825040	C	T	C	Distal Intergenic	FAT3
**6**	chr2:838734	C	C	T	Promoter	CSPG5
**7**	chr2:14869361	C	T	C	Distal Intergenic	JCAD
**8**	chr2:96957597	C	T	C	Distal Intergenic	NA
**9**	chr2:149340388	C	C	A	Intron	NA
**10**	chr2:149406497	C	T	C	Intron	NA
**11**	chr2:149474972	C	T	C	Intron	PUF60
**12**	chr3:11636300	C	C	T	Distal Intergenic	PCSK2
**13**	chr3:71690595	C	C	T	Intron	PNISR
**14**	chr4:1047462	C	C	T	Intron	NA
**15**	chr4:1199481	C	C	G	Intron	MIR6658
**16**	chr4:2363997	C	C	T	Distal Intergenic	NLGN3
**17**	chr4:4406817	C	T	C	Intron	CD40LG
**18**	chr4:11460057	C	C	T	Distal Intergenic	NA
**19**	chr4:16629693	C	C	A	Promoter	SLC25A5
**20**	chr4:25535372	C	T	C	Intron	HPF1
**21**	chr4:47180074	C	T	C	Distal Intergenic	WDFY3
**22**	chr4:69253701	C	C	T	Promoter	LOC112532444
**23**	chr5:361305	C	T	C	Promoter	FNBP4
**24**	chr5:361309	C	T	C	Promoter	FNBP4
**25**	chr5:21620902	C	C	T	Intron	HSD17B12
**26**	chr5:24407778	C	C	T	Promoter	DLL4
**27**	chr6:24291131	C	C	T	Promoter	CFAP58
**28**	chr6:33483099	C	C	T	Distal Intergenic	MKI67
**29**	chr7:2790218	C	C	T	Promoter	MYL1
**30**	chr8:5432804	C	C	T	Distal Intergenic	NA
**31**	chr8:5620269	C	T	C	Promoter	RNASEL
**32**	chr8:6077860	C	T	C	Distal Intergenic	CEP350
**33**	chr8:24217336	C	C	T	Intron	OSBPL9
**34**	chr9:3572449	C	C	T	Distal Intergenic	NA
**35**	chr9:12701729	C	T	C	Promoter	APOD
**36**	chr10:19329800	C	T	C	Intron	GLCE
**37**	chr10:19739557	C	C	T	Distal Intergenic	FAM96A
**38**	chr10:19937743	C	T	C	Intron	WDR76
**39**	chr11:19633168	C	C	T	Distal Intergenic	NA
**40**	chr12:2518820	C	T	C	Promoter	NA
**41**	chr13:1075615	C	C	T	Distal Intergenic	NA
**42**	chr13:2113253	C	T	C	Distal Intergenic	MIR1702
**43**	chr13:10036027	C	A	C	Promoter	SFXN1

**44**	chr13:13645610	C	T	C	Distal Intergenic	ADAMTS2
**45**	chr13:14534988	C	C	T	Promoter	KLHL3
**46**	chr13:14579570	C	T	C	Intron	KLHL3
**47**	chr14:2950462	C	C	T	Intron	MAD1L1
**48**	chr14:5204481	C	T	C	Distal Intergenic	EPN2
**49**	chr14:6284435	C	T	C	Intron	SLC9A3R2
**50**	chr14:11556127	C	T	C	Distal Intergenic	IL20RB
**51**	chr14:12223310	C	T	C	Intron	AXIN1
**52**	chr15:9175581	C	C	T	Intron	SPPL3
**53**	chr15:10441720	C	T	C	Intron	NA
**54**	chr15:12552211	C	T	C	Intron	NA
**55**	chr17:7464339	C	T	C	Intron	NA
**56**	chr17:7503534	C	T	C	Intron	NA
**57**	chr18:9303953	C	T	C	Intron	NA
**58**	chr20:10687800	C	C	T	Promoter	WFDC2L
**59**	chr21:463033	C	G	C	Distal Intergenic	CAMTA1
**60**	chr21:619742	C	T	C	Promoter	NA
**61**	chr21:688182	C	C	A	Intron	NPHP4
**62**	chr28:1385312	C	T	C	Promoter	NA

Number of C	62	29	33
Number of T	0	31	25
Percentage of C	100.0%	48.3%	56.9%
