# Supplementary material for: Genetic and neuro-epigenetic effects of divergent artificial selection for feather pecking behaviour in chickens
Source: BMC Genomics. 2024 Dec 19;25:1219. doi: 10.1186/s12864-024-11137-w (PMC11657628; doi:10.1186/s12864-024-11137-w)
Supplement: Supplementary file 11 — Supplementary Material 11: Additional File 11 Take ESM 11 [file 12864_2024_11137_MOESM11_ESM.pdf]

Supplementary Table S3: Summary of CpG gains between LFP and HFP animals

	SNP Position	Reference	HFP Most Frequent Allele	LFP Most Frequent Allele	Functional Annotation	Gene
**1**	chr1:87002807	T	T	C	Distal Intergenic	CBLB
**2**	chr2:78408648	T	C	T	Distal Intergenic	ANKRD33B
**3**	chr2:99738795	A	C	A	Distal Intergenic	LAMA1
**4**	chr3:15068924	T	C	T	Distal Intergenic	BMP2
**5**	chr3:71792365	T	T	C	Distal Intergenic	FAXC
**6**	chr3:77545754	T	T	C	Distal Intergenic	TBX18
**7**	chr5:24443870	T	C	T	Distal Intergenic	NA
**8**	chr5:37694563	T	T	C	Distal Intergenic	ZNF410
**9**	chr5:55494514	T	T	C	Distal Intergenic	NA
**10**	chr7:35989796	T	C	T	Distal Intergenic	NA
**11**	chr8:5143880	T	T	C	Distal Intergenic	RXRG
**12**	chr8:5145917	T	T	C	Distal Intergenic	RXRG
**13**	chr8:27836602	G	G	C	Distal Intergenic	FOXD3
**14**	chr9:14090220	T	T	C	Distal Intergenic	P3H2
**15**	chr9:14090249	T	T	C	Distal Intergenic	P3H2
**16**	chr9:14877003	T	C	T	Distal Intergenic	SST
**17**	chr12:18193007	T	T	C	Distal Intergenic	NA
**18**	chr12:18596350	T	T	C	Distal Intergenic	NA
**19**	chr13:1897361	T	T	C	Distal Intergenic	HDAC3
**20**	chr13:2089538	T	C	T	Distal Intergenic	MIR1702
**21**	chr13:2089560	T	C	T	Distal Intergenic	MIR1702
**22**	chr14:7189183	T	T	C	Distal Intergenic	HS3ST4
**23**	chr21:462933	T	T	C	Distal Intergenic	CAMTA1
**24**	chr28:2219879	T	C	T	Distal Intergenic	NR1D1
**25**	chr28:4786822	T	C	T	Distal Intergenic	NA
**26**	chr10:19753562	T	C	T	Downstream	FAM96A
**27**	chr20:11388591	T	T	C	Downstream	NA
**28**	chr2:149406250	T	C	T	Exon	NA
**29**	chr18:4710902	T	C	T	Exon	UNC13D
**30**	chr1:14110344	T	T	C	Intron	ATXN7L1
**31**	chr1:195929230	T	T	C	Intron	CHRDL2
**32**	chr2:90498185	T	C	T	Intron	NA
**33**	chr4:17481020	T	T	C	Intron	PASD1
**34**	chr4:69645751	A	A	C	Intron	SMIM14
**35**	chr5:21817998	T	C	T	Intron	NA
**36**	chr5:37703833	T	T	C	Intron	ZNF410
**37**	chr5:58418652	T	C	T	Intron	FERMT2
**38**	chr13:6756471	T	C	T	Intron	NA
**39**	chr14:2878952	T	T	C	Intron	ELFN1
**40**	chr19:8191401	T	C	T	Intron	DHRS11
**41**	chr20:10635671	G	G	C	Intron	NA
**42**	chr21:361391	T	C	T	Intron	VAMP3
**43**	chr21:690769	T	T	C	Intron	NPHP4

**44**	chr1:23414609	G	G	C	Promoter	CPED1
**45**	chr2:15950660	G	G	C	Promoter	MASTL
**46**	chr2:47027018	T	T	C	Promoter	EEPD1
**47**	chr5:58241261	T	T	C	Promoter	NID2
**48**	chr8:3872896	T	T	C	Promoter	NOS1AP
**49**	chr9:12701712	T	C	T	Promoter	APOD
**50**	chr10:20099941	T	C	T	Promoter	NA
**51**	chr12:7051566	T	C	T	Promoter	CACNA1D
**52**	chr14:12336151	T	C	T	Promoter	NA
**53**	chr15:10916349	T	C	T	Promoter	NA
**54**	chr16:274442	T	T	C	Promoter	NA
**55**	chr20:10683918	T	C	T	Promoter	WFDC2L
**56**	chr27:4420187	T	T	C	Promoter	CISD3

Number of C	0	25	31
Number of T	50	26	24
Percentage of C	0.0%	49.0%	56.4%
