# Supplementary material for: Genetic and neuro-epigenetic effects of divergent artificial selection for feather pecking behaviour in chickens
Source: BMC Genomics. 2024 Dec 19;25:1219. doi: 10.1186/s12864-024-11137-w (PMC11657628; doi:10.1186/s12864-024-11137-w)
Supplement: Supplementary file 12 — Supplementary Material 12: Additional File 12 Take ESM 12 [file 12864_2024_11137_MOESM12_ESM.pdf]

Supplementary Table S7: CNV gains in each lineage with changes above 8-fold

	Chr	Start	End	Size	Log2	p.value	Functional Annotation	Distance to TSS	Gene Symbol	Gene Name
HFP Gain > 8X	4	56809748	56816568	6821	8.656709	1.27E-99	Promoter (<=1kb)	0	ARSJ	arylsulfatase family member J
	7	31294424	31302948	8525	8.615352	1.11E-125	Intron (ENSGALT00000020191.5/ENSGALG00000012362.5, intron 16 of 20)	-76021	LOC771456	histamine N-methyltransferase-like
	3	102062154	102068972	6819	8.52635	1.29E-69	Promoter (2-3kb)	-2326	PUM2	pumilio RNA binding family member 2
	1	165868368	165878598	10231	8.515599	0	Promoter (<=1kb)	0	MTRF1	mitochondrial translation release factor 1
	3	27700284	27708808	8525	8.456909	1.58E-86	Intron (ENSGALT00000016321.5/ENSGALG00000010043.6, intron 7 of 7)	11903	NA	NA
Largest HFP Gain	5	10172884	10196752	23869	0.8357636	1.09E-66	Promoter (<=1kb)	282	RIC3	RIC3 acetylcholine receptor chaperone
LFP Gain > 8X	1	16131858	16140382	8525	-10.15961	0	3' UTR	22723	NA	NA
	13	15093514	15100332	6819	-10.14482	1.15E-261	Exon (ENSGALT00000027784.5/ENSGALG00000006297.6, exon 5 of 12)	14992	TRPC7	transient receptor potential cation channel subfamily C member 7
	2	80681454	80688272	6819	-10.07366	1.00E-79	Distal Intergenic	-62158	VWC2	von Willebrand factor C domain containing 2
	2	88284048	88292572	8525	-9.363117	4.60E-99	Intron (ENSGALT00000046850.1/ENSGALG00000013200.5, intron 1 of 18)	35941	GABBR2	gamma-aminobutyric acid type B receptor subunit 2
	9	4440674	4447492	6819	-9.193936	7.24E-183	Promoter (<=1kb)	336	SENP2	SUMO1/sentrin/SMT3 specific peptidase 2
	2	11627248	11634068	6821	-8.690841	1.72E-79	Distal Intergenic	-142900	PITRM1	pitrilysin metallopeptidase 1
	4	89131434	89138252	6819	-8.570111	3.76E-110	5' UTR	17228	PTPRA	protein tyrosine phosphatase, receptor type A
	4	74031954	74042182	10229	-8.132569	6.36E-164	3' UTR	12199	SEPSECS	Sep (O-phosphoserine) tRNA:Sec (selenocysteine) tRNA synthase
Largest LFP Gain	4	25666218	25688382	22165	-1.877699	5.20E-175	Promoter (<=1kb)	0	SH3RF1	SH3 domain containing ring finger 1
