# Supplementary material for: Genetic and neuro-epigenetic effects of divergent artificial selection for feather pecking behaviour in chickens
Source: BMC Genomics. 2024 Dec 19;25:1219. doi: 10.1186/s12864-024-11137-w (PMC11657628; doi:10.1186/s12864-024-11137-w)
Supplement: Supplementary file 13 — Supplementary Material 13: Additional File 13 Take ESM 13 [file 12864_2024_11137_MOESM13_ESM.pdf]

Supplementary Table S11: Overview of overlaps among the omic levels investigated here and publicly available QTLs for feather pecking

Overlap number	Entity	Chromosome	Start	End	Length (bp)
1	SNP	6	10,931,835	10,931,835	1
	SNP	6	11,352,053	11,352,053	1
	SNP	6	11,440,005	11,440,005	1
	SNP	6	11,440,027	11,440,027	1
	QTL	6	7,996,326	12,167,292	4,170,967
	DMR	6	8,241,601	8,241,900	300
	DMR	6	8,241,901	8,242,200	300
	CNV	6	8,031,404	8,038,222	6,819
	CNV	6	8,045,044	8,053,568	8,525
	CNV	6	8,092,784	8,099,602	6,819
	CNV	6	8,109,834	8,120,062	10,229
	CNV	6	8,249,644	8,256,462	6,819
	CNV	6	8,580,414	8,587,232	6,819
	CNV	6	8,672,484	8,682,712	10,229
	CNV	6	8,919,708	8,931,642	11,935
	CNV	6	9,197,624	9,204,442	6,819
	CNV	6	9,277,758	9,286,282	8,525
	CNV	6	9,611,938	9,618,758	6,821
	CNV	6	9,802,898	9,811,422	8,525
	CNV	6	10,138,784	10,145,602	6,819
	CNV	6	10,155,834	10,162,652	6,819
	CNV	6	10,177,998	10,186,522	8,525
	CNV	6	10,249,608	10,256,428	6,821
	CNV	6	10,276,888	10,283,708	6,821
	CNV	6	10,392,828	10,399,648	6,821
	CNV	6	10,428,634	10,435,452	6,819
	CNV	6	10,542,868	10,551,392	8,525
	CNV	6	10,708,254	10,715,072	6,819
	CNV	6	10,894,098	10,900,918	6,821
	CNV	6	10,948,658	10,957,182	8,525
	CNV	6	11,037,318	11,044,138	6,821
	CNV	6	11,102,108	11,108,928	6,821
	CNV	6	11,258,968	11,265,788	6,821
	CNV	6	11,352,744	11,362,972	10,229
	CNV	6	11,449,928	11,456,748	6,821
	CNV	6	11,588,034	11,596,558	8,525
	CNV	6	11,678,398	11,685,218	6,821
	CNV	6	11,915,394	11,923,918	8,525
	CNV	6	11,946,084	11,952,902	6,819
	CNV	6	12,123,404	12,130,222	6,819
2	SNP	21	619,742	619,742	1
	DMR	21	619,501	619,800	300
3	QTL	21	1,929,204	1,929,244	41
	CNV	21	1,929,208	1,937,732	8,525
4	QTL	19	4,226,903	4,226,943	41
	CNV	19	4,220,728	4,229,252	8,525

5	QTL	9	15,790,849	15,790,889	41
	CNV	9	15,787,448	15,795,972	8,525
6	QTL	9	15,294,750	15,294,790	41
	CNV	9	15,294,704	15,301,522	6,819
7	QTL	5	37,432,740	37,432,780	41
	CNV	5	37,432,424	37,439,242	6,819
8	QTL	5	23,404,043	23,404,083	41
	CNV	5	23,398,568	23,407,092	8,525
9	QTL	5	10,936,584	10,936,624	41
	CNV	5	10,931,608	10,938,428	6,821
10	QTL	4	52,221,679	52,221,719	41
	CNV	4	52,218,184	52,225,002	6,819
11	QTL	3	104,800,409	104,800,449	41
	CNV	3	104,793,564	104,802,088	8,525
12	QTL	3	18,739,559	18,739,599	41
	CNV	3	18,735,394	18,742,212	6,819
13	QTL	1	112,894,891	112,894,931	41
	CNV	1	112,887,198	112,895,722	8,525
14	DMR	z	310,801	311,100	300
	DMR	z	311,101	311,400	300
	CNV	z	304,344	311,162	6,819
15	DMR	26	4,155,001	4,155,300	300
	DMR	26	4,155,301	4,155,600	300
	CNV	26	4,152,528	4,159,348	6,821
16	DMR	18	48,301	48,600	300
	CNV	18	38,364	50,298	11,935
17	DMR	15	11,257,801	11,258,100	300
	DMR	15	11,258,101	11,258,400	300
	CNV	15	11,255,558	11,265,788	10,231
18	DMR	6	35,197,501	35,197,800	300
	CNV	6	35,197,168	35,203,988	6,821
19	DMR	5	23,576,401	23,576,700	300
	DMR	5	23,576,701	23,577,000	300
	CNV	5	23,574,184	23,582,708	8,525
20	DMR	1	89,537,101	89,537,400	300
	DMR	1	89,537,401	89,537,700	300
	DMR	1	89,537,701	89,538,000	300
	CNV	1	89,532,108	89,544,042	11,935
21	SNP	21	676,303	676,303	1
	SNP	21	676,312	676,312	1
	SNP	21	676,319	676,319	1
	CNV	21	670,918	677,738	6,821
22	SNP	20	12,819,780	12,819,780	1
	SNP	20	12,819,784	12,819,784	1
	CNV	20	12,819,044	12,825,862	6,819
23	SNP	20	11,704,085	11,704,085	1
	CNV	20	11,702,268	11,710,792	8,525

24	SNP	18	3,794,693	3,794,693	1
	CNV	18	3,792,774	3,801,298	8,525
25	SNP	15	11,481,594	11,481,594	1
	CNV	15	11,480,618	11,487,438	6,821
26	SNP	15	6,021,039	6,021,039	1
	CNV	15	6,016,094	6,022,912	6,819
27	SNP	8	5,620,269	5,620,269	1
	SNP	8	5,620,311	5,620,311	1
	CNV	8	5,618,828	5,625,648	6,821
28	SNP	6	29,210,342	29,210,342	1
	CNV	6	29,209,208	29,217,732	8,525
29	SNP	5	55,494,514	55,494,514	1
	CNV	5	55,490,078	55,496,898	6,821
30	SNP	5	53,643,119	53,643,119	1
	CNV	5	53,640,154	53,646,972	6,819
31	SNP	4	85,357,565	85,357,565	1
	CNV	4	85,354,858	85,361,678	6,821
32	SNP	3	105,450,463	105,450,463	1
	CNV	3	105,449,988	105,456,808	6,821
33	SNP	2	88,155,991	88,155,991	1
	SNP	2	88,156,009	88,156,009	1
	CNV	2	88,154,468	88,161,288	6,821
34	SNP	1	131,845,509	131,845,509	1
	CNV	1	131,838,274	131,846,798	8,525
35	SNP	1	103,616,588	103,616,588	1
	CNV	1	103,615,408	103,623,932	8,525
36	SNP	1	66,580,310	66,580,310	1
	CNV	1	66,575,988	66,582,808	6,821
37	SNP	1	51,507,306	51,507,306	1
	CNV	1	51,505,494	51,515,722	10,229
38	SNP	1	20,808,614	20,808,614	1
	CNV	1	20,798,444	20,810,378	11,935
